# Polysaccharide II Surface Anchoring, the Achilles’ Heel of Clostridioides difficile

**DOI:** 10.1128/spectrum.04227-22

**Published:** 2023-02-23

**Authors:** Jeanne Malet-Villemagne, Liang Yucheng, Laurent Evanno, Sandrine Denis-Quanquin, Jean-Emmanuel Hugonnet, Michel Arthur, Claire Janoir, Thomas Candela

**Affiliations:** a Micalis Institute, Université Paris-Saclay, INRAE, AgroParisTech, Jouy-en-Josas, France; b Biomolécules: Conception, Isolement et Synthèse (BioCIS), Université Paris-Saclay, CNRS, Orsay, France; c Laboratoire de Chimie de l'ENS Lyon, CNRS and Université de Lyon, Lyon, France; d INSERM UMR-S 1138, Centre de Recherche des Cordeliers, Sorbonne Université, Université Paris Cité, Paris, France; The University of Tennessee Knoxville

**Keywords:** *Clostridium difficile*, essentiality, pathogens, peptidoglycan, polysaccharides, polysaccharide anchoring, surface structures

## Abstract

Cell wall glycopolymers (CWPGs) in Gram-positive bacteria have been reported to be involved in several bacterial processes. These polymers, pillars for proteins and S-layer, are essential for the bacterial surface setup, could be essential for growth, and, in pathogens, participate most often in virulence. CWGPs are covalently anchored to peptidoglycan by proteins that belong to the LytR-CpsA-PSr (LCP) family. This anchoring, important for growth, was reported as essential for some bacteria such as Bacillus subtilis, but the reason why CWGP anchoring is essential remains unknown. We studied LcpA and LcpB of Clostridioides difficile and showed that they have a redundant activity. To delete both *lcp* genes, we set up the first conditional-lethal mutant method in C. difficile and showed that polysaccharide II (PSII) anchoring at the bacterial surface is essential for C. difficile survival. In the conditional-lethal mutant, C. difficile morphology was impaired, suggesting that peptidoglycan synthesis was affected. Because Lcp proteins are transferring CWPGs from the C_55_-undecaprenyl phosphate (also needed in the peptidoglycan synthesis process), we assumed that there was competition between PSII and peptidoglycan synthesis pathways. We confirmed that UDP-MurNAc-pentapeptide precursor was accumulated, showing that peptidoglycan synthesis was blocked. Our results provide an explanation for the essentiality of PSII anchoring in C. difficile and suggest that the essentiality of the anchoring of CWPGs in other bacteria can also be explained by the blocking of peptidoglycan synthesis. To conclude, our results suggest that Lcps are potential new targets to combat C. difficile infection.

**IMPORTANCE** Cell wall glycopolymers (CWGPs) in Gram-positive bacteria have been reported to be involved in several bacterial processes. CWGP anchoring to peptidoglycan is important for growth and virulence. We set up the first conditional-lethal mutant method in Clostridioides difficile to study LcpA and LcpB involved in the anchoring of CWPGs to peptidoglycan. This study offers new tools to reveal the role of essential genes in C. difficile. LcpA and LcpB activity was shown to be essential, suggesting that they are potential new targets to combat C. difficile infection. In this study, we also showed that there is competition between the polysaccharide II synthesis pathway and peptidoglycan synthesis that probably exists in other Gram-positive bacteria. A better understanding of these mechanisms allows us to define the Lcp proteins as a therapeutic target for potential design of novel antibiotics against pathogenic Gram-positive bacteria.

## INTRODUCTION

In recent years, there has been an increasing interest in cell wall glycopolymers (CWGPs) in Gram-positive bacteria for their role in bacterial physiology and pathogenicity. These polymers represent up to 50% of the dry weight of the cell wall (1). CWGPs are covalently linked to peptidoglycan (PG) and support surface proteins (like Cwp in Clostridioides difficile or SLH in Bacillus subtilis) that are noncovalently anchored at the very end of CWGPs (2, 3). Most CWGPs are essential and are involved in cell division and cell shape maintenance (for review, see reference 4). In pathogens, they are essential for virulence (5–11) and are therefore thought to be targets for the development of new antimicrobial molecules (12, 13).

CWGP anchoring is of major importance for the physiology of Gram-positive bacteria. This process was shown to be performed by proteins belonging to the LytR-CpsA-PSr (LCP) family. Members of this protein family are widespread in the bacterial world, as they were identified in eight different bacterial phyla (14). The Lcp enzymes transfer CWGPs from a lipid carrier, the C_55_-undecaprenyl phosphate (C_55_P), to the nascent or mature PG (15–19). Lcp proteins are therefore key players in bacterial surface assembly. In Gram-positive bacteria, several copies of *lcp* genes are often observed, but the gene products can have distinct catalytic activities. For example, in B. subtilis, three Lcp proteins have been identified, and one of them (TagU) has stronger activity than the other two (20). In Mycobacterium tuberculosis, the only Lcp is essential (21). Finally, in Staphylococcus aureus, LcpA is the main transferase of teichoic acids, whereas LcpC is dedicated to capsular polysaccharide transfer (19). The Lcp proteins are at least partially redundant in B. subtilis (22), Staphylococcus aureus (23, 24), and Streptococcus pneumoniae (25, 26). Additionally, the Lcp proteins are good targets for the development of specific inhibitors because their soluble catalytic domain is extracellular (accessible to immune system cells), and they have no homologs in mammals (14). In C. difficile, Lcp proteins were studied individually (27), but their redundancy and essentiality for survival have never been assessed experimentally.

Clostridioides difficile (previously known as Clostridium difficile) is a Gram-positive, motile, strictly anaerobic, and spore-forming enteric pathogen. C. difficile infections (CDIs) are a primary cause of nosocomial diarrhea and antibiotic-associated colitis (28). Additionally, the numbers of incidences and severe clinical forms have been increasing in recent years due to emerging hypervirulent and antibiotic-resistant strains (including C. difficile ribotype 027) (29). Consequently, C. difficile is considered an urgent threat to public health by the Centers for Disease Control and prevention (30). Moreover, multiple resistance mechanisms to currently used antibiotics, often mediated by plasmid acquisition (31, 32), are observed and cause concerns about future treatment options for CDI management. Therefore, developing new strategies and discovering new bacterial targets is necessary.

In C. difficile, three CWPGs have been identified: two teichoic acids (TAs) anchored in the peptidoglycan (polysaccharides I and II [PSI and PSII]) (33) and one lipoteichoic acid (LTA) anchored directly in the membrane (34). Only PSII and the LTA are conserved among C. difficile strains. All biosynthesis genes of these glycopolymers are encoded in a single locus of the chromosome, and all genes predicted to encode enzymes involved in PSII synthesis are reported to be essential for bacterial survival (3, 35), but surprisingly not the genes encoding PSII-anchoring proteins (*lcpA* and *lcpB*) (27). Note that Cwp proteins, including the S-layer protein SlpA, are noncovalently linked to PSII (3).

In this study, we focused on PSII. PSII biosynthesis is predicted to be initiated by the transferase CD2783, transferring the first sugar unit (UDP-glucose) on the C_55_P lipid carrier. Then, several cytoplasmic glycosyltransferases catalyze the transfer of sugar units on the chain. When finished, the chain is flipped outside the cell by the flippase MviN (27) and transferred to the preexisting chains to form a C_55_P-polymerized PSII molecule. PSII is then transferred from the lipid carrier to the PG by LcpA and LcpB (27).

In this work, we studied the two Lcps of C. difficile to evaluate the importance of a correct polysaccharide anchoring in the physiology of the bacterium. We wanted to determine if the *lcp* genes were essential for survival.

## RESULTS

### Construction of *lcp* single mutants.

To facilitate the screen of the allelic exchange technique in C. difficile (36), we decided to replace the open reading frame (ORF) of *lcpA* and *lcpB* with the ORF of the *catP* gene expressing thiamphenicol (Th) resistance. We therefore constructed the pJV10 vector that harbors an *ermB* gene (Fig. S1 in the supplemental material) conferring erythromycin (Er) resistance. In the genome of the 630 strain, two copies of the *ermB* gene are found. In contrast, 630Δ*erm* harbors only one *ermB* gene (37), which is usually not expressed. However, we were unable to conjugate the pJV10 plasmid in the 630Δ*erm* that became, under these conditions, resistant to erythromycin, probably because the remaining *ermB* gene was sufficiently expressed during the conjugation process. Therefore, we chose to construct a “true” 630Δ*erm* by deleting both *ermB* genes directly from the clinical 630 strain and obtained the JMV1 strain.

We first deleted *lcpA* and *lcpB* separately in the JMV1 strain using the allelic exchange method (36). Thanks to the presence of the *catP* gene, after selection of the first crossover (36), a simple restreak on a petri dish in the presence of thiamphenicol allowed the identification of potential mutant clones. The JMV3 (Δ*lcpA*) strain was easily obtained (21 mutant clones were thiamphenicol resistant out of 25), whereas the JMV4 (Δ*lcpB*) strain was quite hard to get (3 mutant clones were thiamphenicol resistant out of 187), suggesting that *lcpB* plays an important role for C. difficile growth.

### *lcpA* and *lcpB* are redundant.

**FIG 1:**
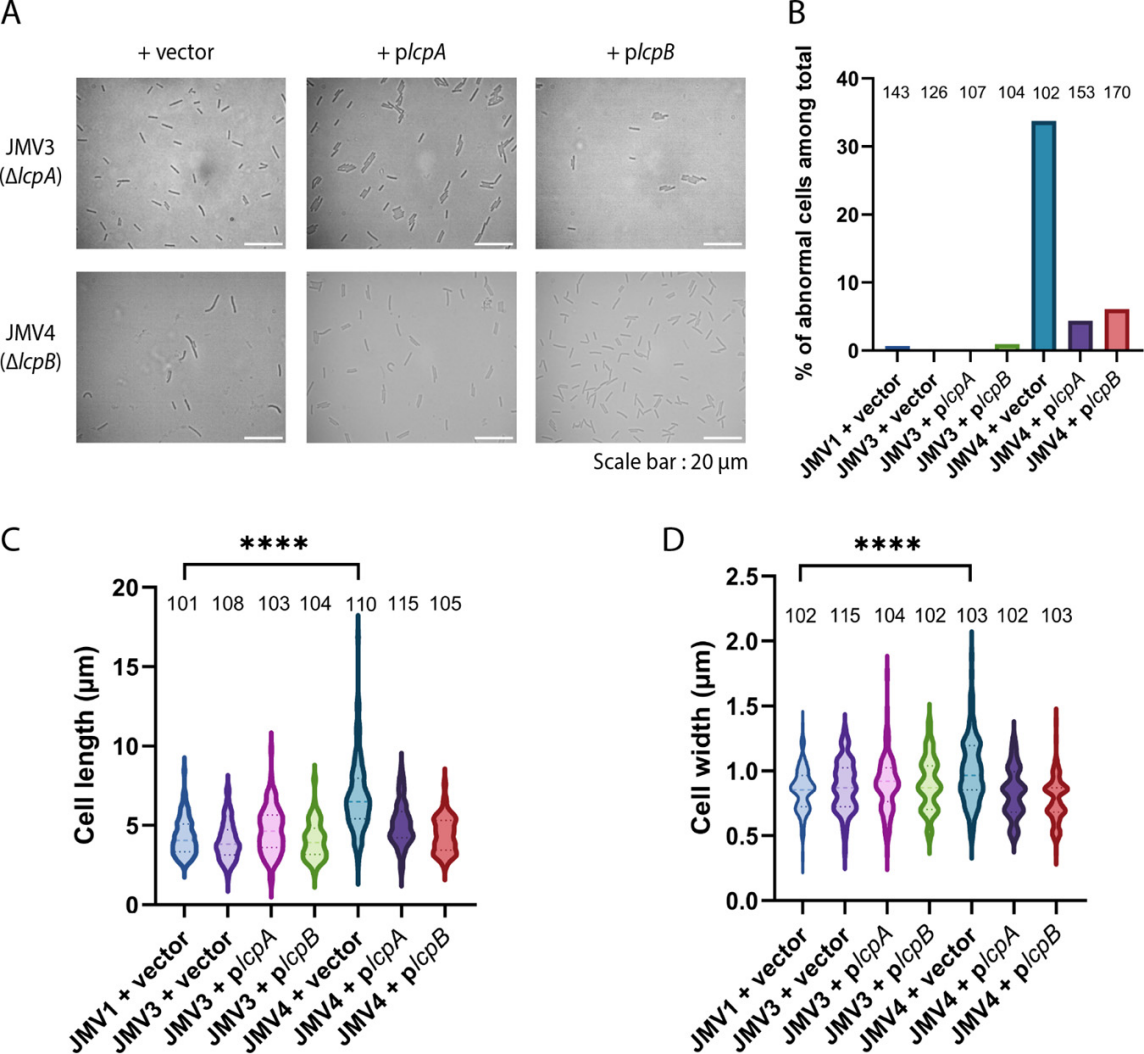
The Δ*lcpB* mutant (JMV4) presents curved, inflated, longer, and larger cells than the parental strain JMV1. (A) JMV3 (**Δ***lcpA*) and JMV4 (**Δ***lcpB*) single mutants were analyzed by optical microscopy and complemented with pMTL84222 (vector), p*lcpA* (plasmid carrying *lcpA* expressed with its own promoter), or p*lcpB* (plasmid carrying *lcpB* expressed with its own promoter); scale bar, 20 μm. (B) The percentage of abnormal (curved, thick, or inflated) cells among total cells was calculated by measuring more than 100 cells for each strain. (C) Cell length of the parental strain JMV1, JMV3, and JMV4 complemented with the vector (pMTL84222), p*lcpA*, or p*lcpB*. (D) Cell width of the parental strain JMV1, JMV3, and JMV4 complemented with the vector (pMTL84222), p*lcpA*, or p*lcpB*. For B to D, the number above each bar represents the number of cells counted. Data were analyzed by Student’s *t* test; ****, *P* < 0.0001.

We first confirmed that the *lcpB* mutant (JMV4) showed a growth defect (Fig. S2). We then observed the morphology of the mutant cells by classical optical microscopy (Fig. 1A) and measured the cell length and width (Fig. 1C and D), allowing us to determine a percentage of “normal morphology” (Fig. 1B). Contrary to the Δ*lcpA*-mutant bacteria (JMV3) whose morphology is normal, almost 35% of the Δ*lcpB* (JMV4) cells were curved or inflated (Fig. 1; Fig. S3). Cells were also significantly longer and thicker than JMV1 cells (Fig. 1C and D). These observed morphological and growth defects in the absence of *lcpB* confirmed the previous results obtained by Vedantam et al. (27) and suggest that LcpB plays a major role in anchoring PSII to peptidoglycan.

We also observed that bacterial morphology and growth were restored in the strains JMV4 + pMEZ12 (complementation plasmid bearing *lcpA*, named p*lcpA* for simplicity) and JMV4 + pJV21 (complementation plasmid bearing *lcpB*, named p*lcpB* for simplicity) (Fig. 1A; Fig. S2). Indeed, the abnormal cell ratios were reduced to 4% or 6% when complementation with *lcpA* or *lcpB* was introduced, respectively (Fig. 1B). These results suggest that LcpA can compensate for the absence of LcpB in anchoring PSII to peptidoglycan and that both Lcp proteins have redundant functions in C. difficile.

**FIG 2:**
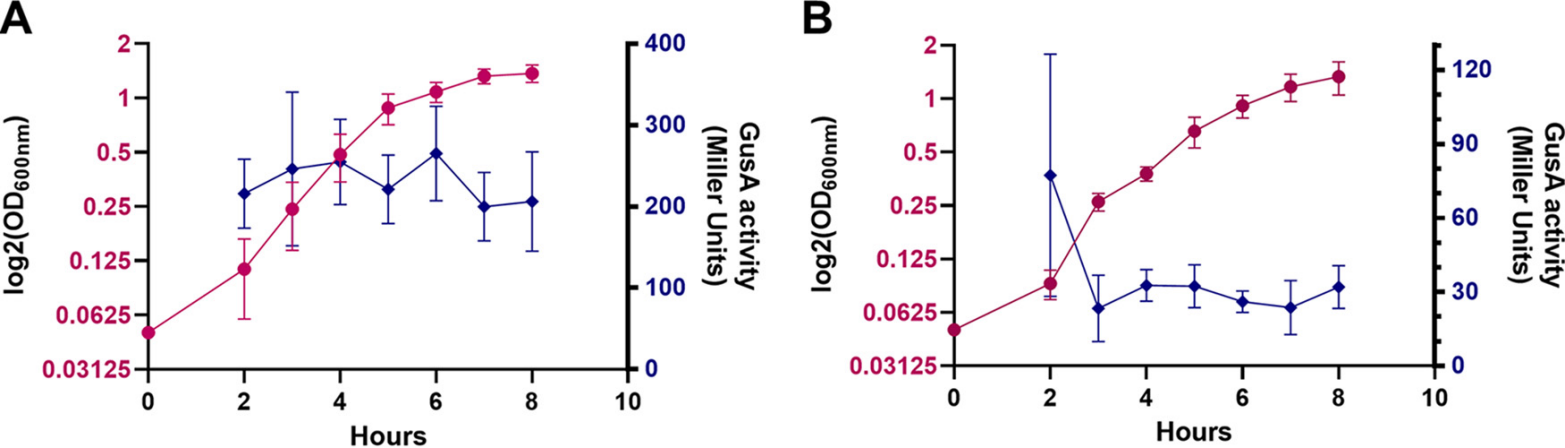
(A and B) Both *lcpA* and *lcpB* are constitutively expressed. GusA activity measured for *lcpA* (A) and *lcpB* (B) promoters is shown. GusA activity (blue curve) was measured in Miller units, and growth (red curve) was followed by measuring the optical density at 600 nm (OD_600 nm_) for 8 h.

Considering the absence of phenotype in the JMV3 strain, we wondered if *lcpA* was expressed, and we assessed the expression of *lcpA* and *lcpB* by measuring promoter activity by β-glucuronidase assay. As shown in Fig. 2A and B, *lcpA* and *lcpB* were constitutively expressed with means of 230 and 35 Miller units, respectively, but *lcpA* was transcribed at a higher level than *lcpB*. This result suggests that even if transcribed at a low level, *lcpB* is important for cell growth and morphology.

### The *ΔlcpA* (JMV3) and *ΔlcpB* (JMV4) mutant strains present a normal surface protein profile but an altered PSII layer.

**FIG 3:**
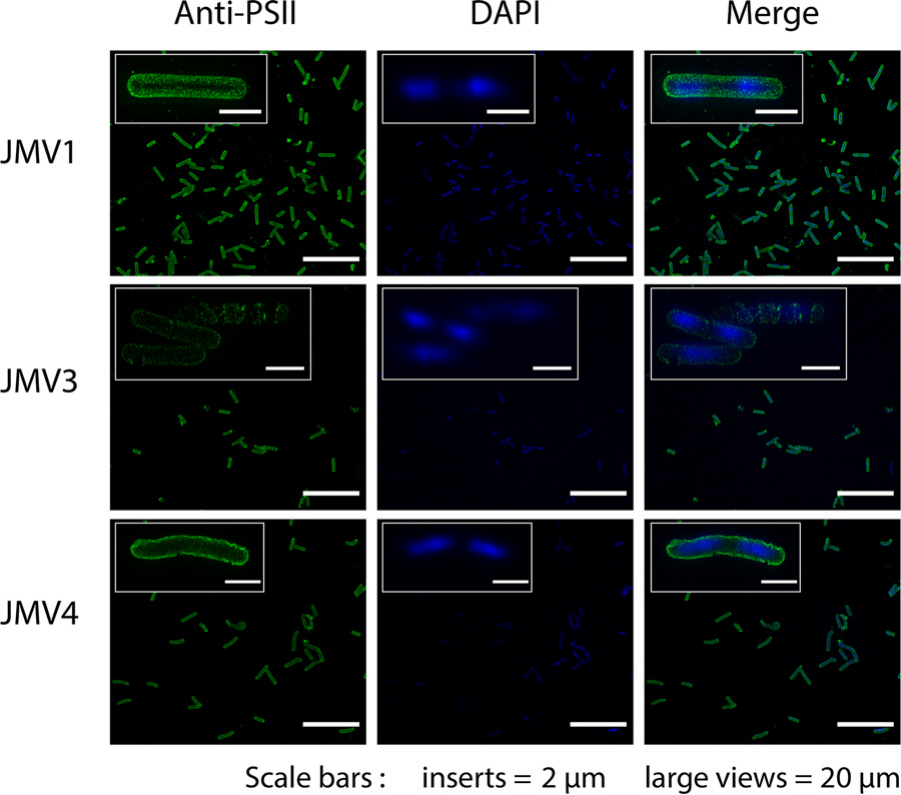
Both Δ*lcpA* and Δ*lcpB* exhibit an altered PSII layer at the surface. An immunofluorescence assay of JMV1, JMV3, and JMV4 strains was performed using a superresolution microscope. Bacteria were stained for DNA (DAPI, blue) and PSII (anti-PSII, green). The merged pictures show both localizations simultaneously; scale bars, 20 μm. Insets show magnifications of the respective images; scale bars, 2 μm.

Because Cwp proteins are noncovalently anchored to PSII, we analyzed the S-layer content (Fig. S4) and found no differences between the CWP proteins of the parental strain and the single mutants. To assess the presence of PSII at the bacterial surface, we purified PSII, checked that it was not contaminated with LTA by nuclear magnetic resonance (NMR) (Fig. S5), and coupled it with bovine serum albumin (BSA). After immunization, we obtained specific antibodies able to recognize the PSII (Fig. S6). Using a superresolution microscope, the JMV1 parental strain showed a homogenous and continuous layer of PSII along the bacterium. In contrast, both JMV3 and JMV4 mutant strains showed an altered PSII layer (Fig. 3). JMV3 cells presented a holed layer of PSII. The JMV4 mutant strain presented a smooth PSII layer. Alterations in PSII deposition at the surface were present in both mutants, but the PSII layer was altered differently in the JMV3 and JMV4 strains, suggesting that even though they are redundant in activity, LcpA and LcpB have slightly different roles in PSII anchoring at the bacterial surface.

### PSII anchoring is essential for C. difficile survival.

Once the single mutants were obtained and their phenotypes confirmed, we tried to get a double mutant strain to assess the essentiality of PSII anchoring for C. difficile survival. The first strategy was to use our improved allelic exchange method using the pJV13 plasmid and the C. difficile JMV1 strain. Despite the facilitated screening of mutants, we failed to isolate a double *lcp* mutant over the 450 clones tested. This result suggested that deleting both *lcpA* and *lcpB* genes was not possible perhaps because of the essentiality of both LcpA and LcpB.

**FIG 4:**
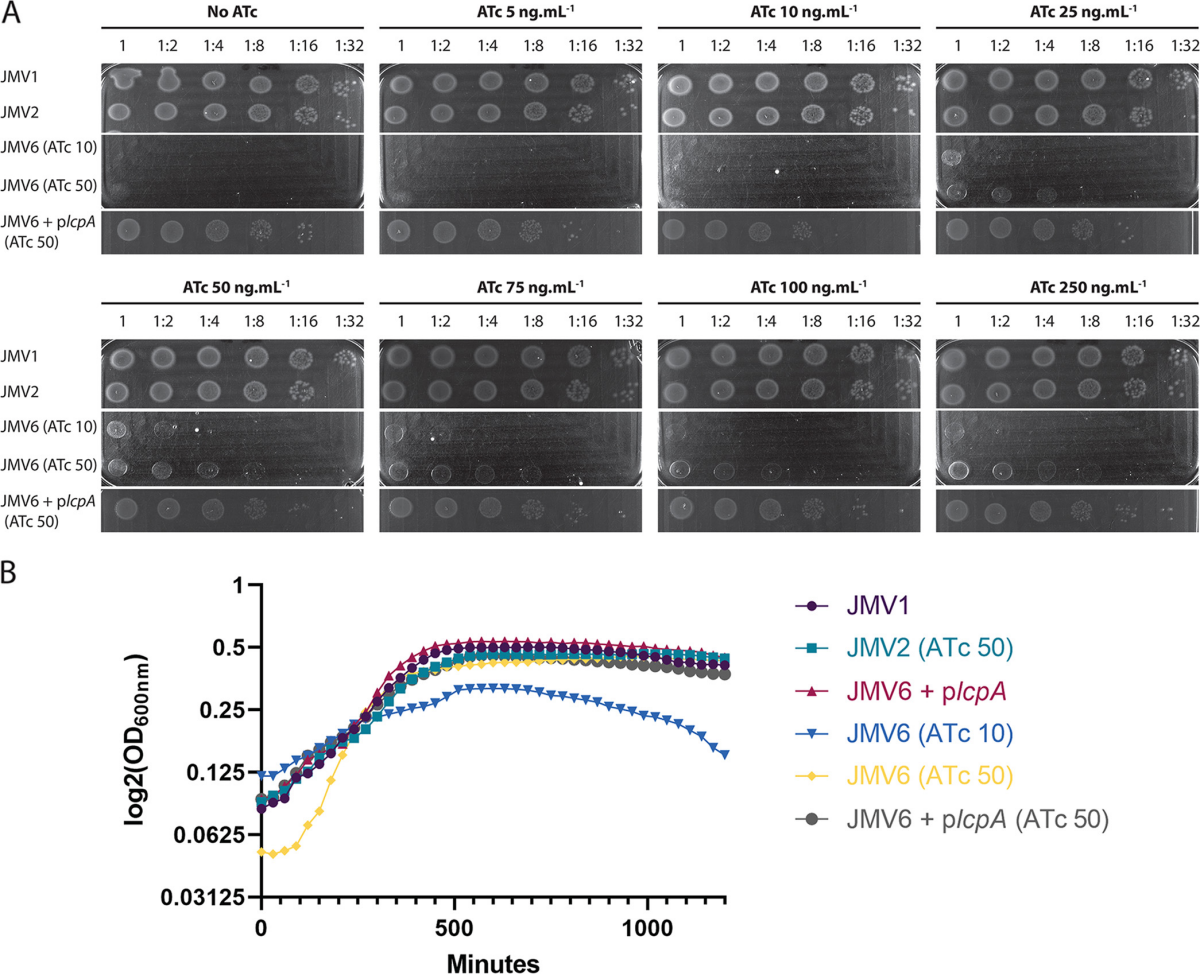
The *lcp* conditional-lethal mutant (JMV6) is not able to grow without ATc induction of the P*_tet_-lcpB* copy. (A) JMV1 and JMV2 grown in liquid culture were diluted and plated on BHI agar petri dishes. JMV6 grown in liquid culture in the presence of 10 (JMV6 [ATC 10]) or 50 ng mL^−1^ ATc (JMV6 [ATc 50]) was diluted and plated on BHI agar petri dishes. The control strain, JMV6 harboring the plasmid p*lcpA* grown in liquid culture in the presence of 50 ng mL^−1^ ATc (JMV6 + p*lcpA* [ATc 50]), was diluted and plated on BHI agar petri dishes. Petri dishes contained ATc from 0 to 250 ng mL^−1^ in the BHI agar medium. (B) JMV1, JMV2, JMV6, and JMV6 + p*lcpA* were grown in the presence of 50 ng mL^−1^ ATc, and growth was measured for 20 h (1,200 min) without ATc (JMV1 and JMV6 + p*lcpA*), in the presence of 10 ng mL^−1^ ATc (JMV6 [ATc 10]), or in the presence of 50 ng mL^−1^ ATc (JMV2 [ATc 50], JMV6 [ATc 50], and JMV6 + p*lcpA* [ATc 50]). The graph represents the mean of three independent experiments.

**FIG 5:**
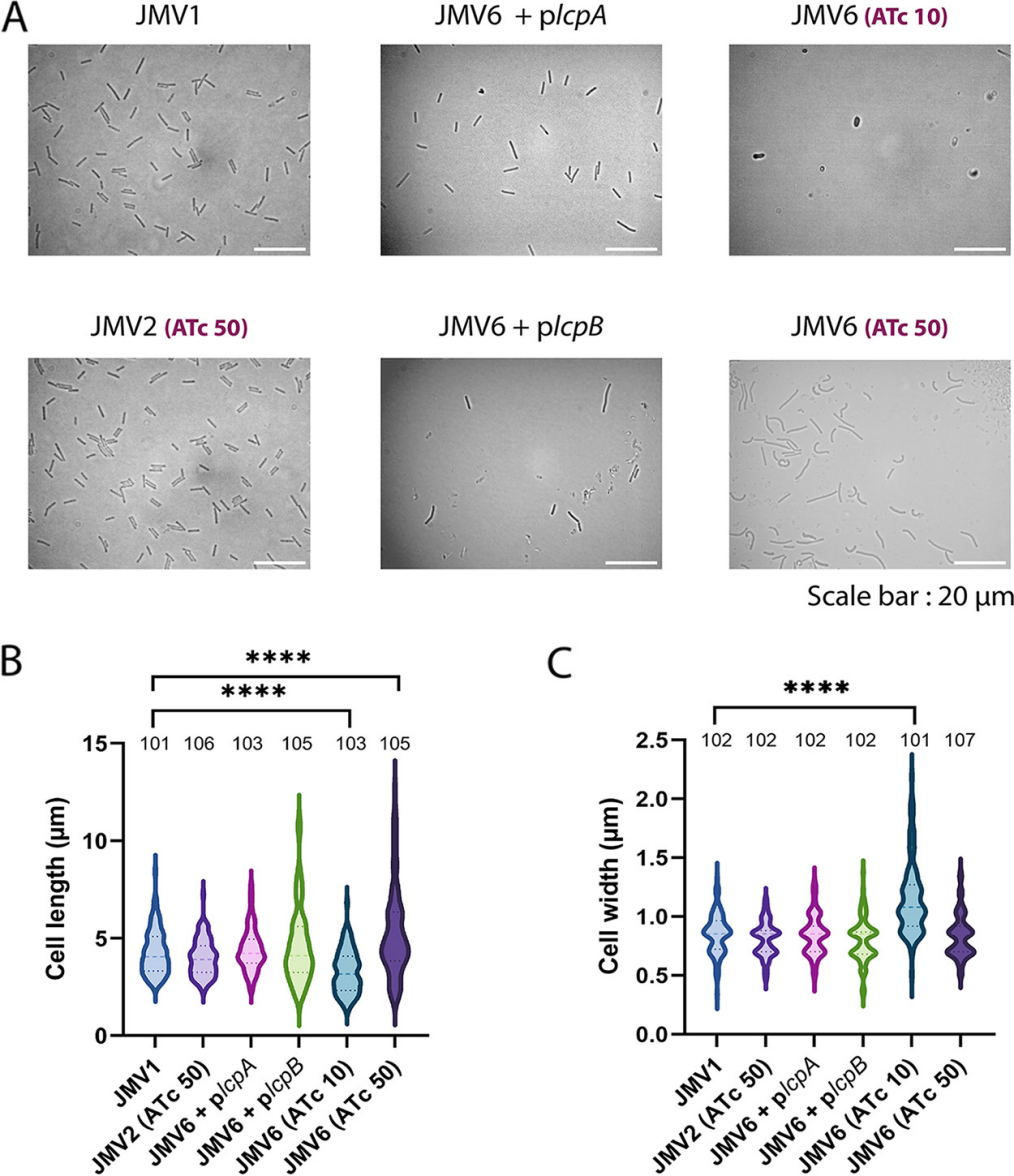
In the presence of 10 ng mL^−1^ ATc, the *lcp* conditional-lethal mutant (JMV6) loses its rod shape. (A) JMV1, JMV6 + p*lcpA*, JMV6 + p*lcpB*, JMV2 in the presence of 50 ng mL^−1^ ATc (JMV2 [ATc 50]), and JMV6 in the presence of 10 (JMV6 [ATc 10]) or 50 ng mL^−1^ ATc (JMV6 [ATc 50]) were observed by optical microscopy; scale bar, 20 μm. (B and C) Cell length (B) and cell width (C) of bacteria from each strain observed in A were measured. The number above each column represents the number of cells counted. Data were analyzed by Student’s *t* test; ****, *P* < 0.0001.

To assess the essentiality of *lcp* genes in C. difficile, we elaborated a new strategy based on the construction of a conditional-lethal mutant (Fig. S7). The first step was to insert an extra copy of *lcpB* under the control of a P*_tet_* promoter in the *ermB* locus of the 630 strain to mimic the JMV1 strain by removing both *ermB* genes, giving rise to the JMV2 strain. The second step was to delete both *lcpA* and *lcpB* in the JMV2 strain using pJV13 plasmid in the presence of 100 ng mL^−1^ anhydrotetracycline (ATc). We obtained the conditional-lethal mutant strain JMV6 that was not able to grow without induction of the additional copy of *lcpB* (Fig. 4A; Fig. S8). To confirm this phenotype, the conditional-lethal mutant strain JMV6 was grown in the presence of 10 or 50 ng mL^−1^ ATc and plated on a petri dish with 0 to 250 ng mL^−1^ ATc (Fig. 4A). No growth was observed on plates at 10 ng mL^−1^ ATc or less, showing that the presence of at least one *lcp* gene is essential for C. difficile growth. In liquid culture, the conditional-lethal mutant strain JMV6 showed impaired growth in the presence of 10 ng mL^−1^ ATc that was restored by adding 50 ng mL^−1^ ATc (Fig. 4B). Without ATc, growth was restored when *lcpA* was present (on p*lcpA* plasmid), confirming the redundancy of Lcp activity. We also assessed morphology using microscopy and confirmed that the conditional-lethal mutant strain (JMV6) grown with 10 ng mL^−1^ ATc had a marked phenotype with ellipsoid cells shorter and thicker than JMV1 cells (Fig. 5). In the presence of 50 ng mL^−1^ ATc, some bacilli were curved and long, but the rod shape was restored with comparable cell width and increased cell length compared to JMV1 cells (Fig. 5). Finally, the addition of p*lcpA* or p*lcpB* fully restored the bacterial shape similar to the controls (JMV1 and JMV2 strains). Our results show that the absence of *lcpA* and *lcpB* is lethal for C. difficile and suggest that PSII anchoring is essential for C. difficile growth. In addition, this result suggests that only *lcpA* and *lcpB* are involved in PSII anchoring.

### PSII remains at the bacterial surface in the JMV6 strain.

**FIG 6:**
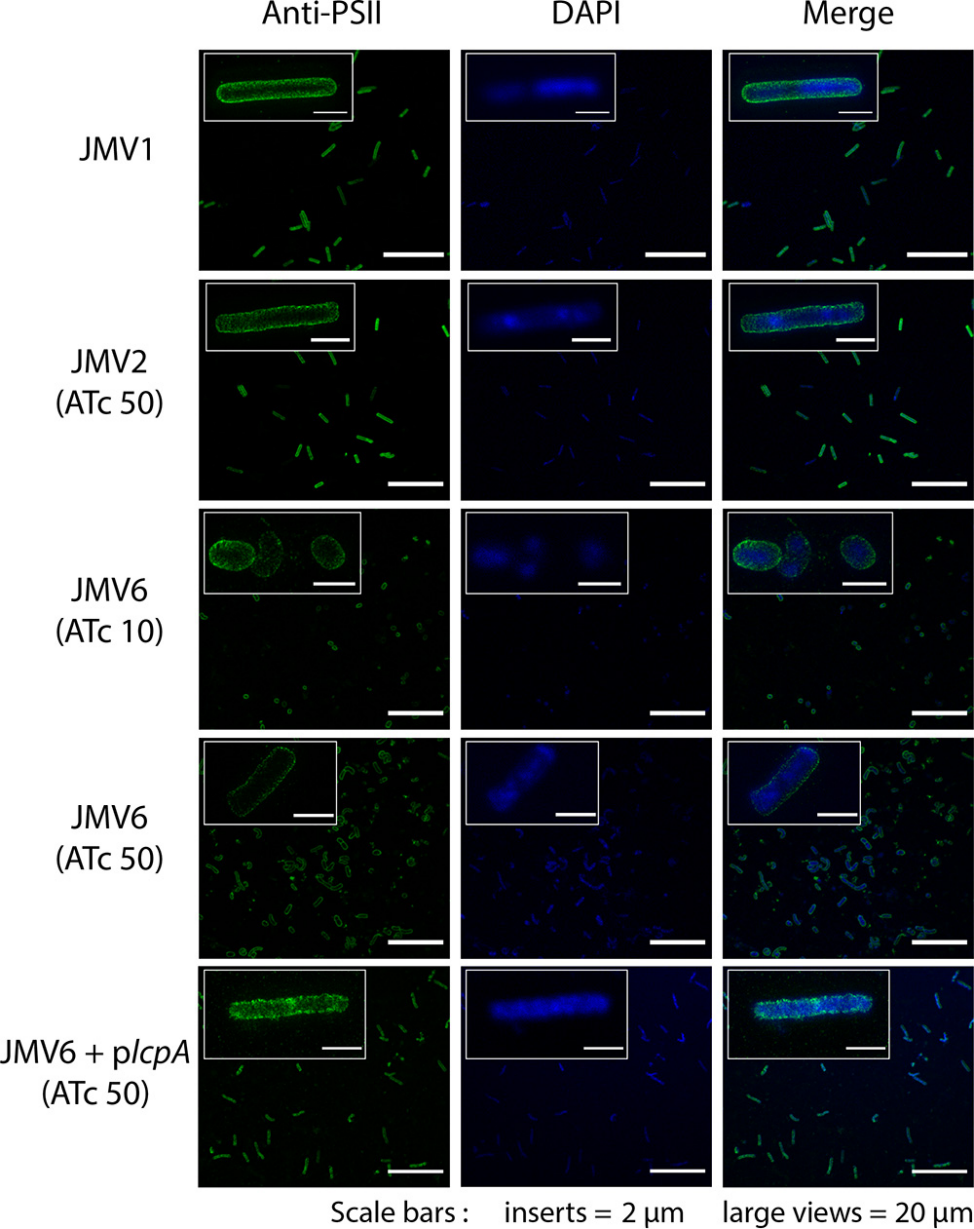
In the presence of 10 ng mL^−1^ ATc, the *lcp* conditional-lethal mutant (JMV6) loses its rod shape, but PSII is still detected at the surface. Immunofluorescence assays of JMV1, JMV2 in the presence of 50 ng mL^−1^ (JMV2 [ATc 50]), JMV6 in the presence of 10 ng mL^−1^ (JMV6 [ATc 10]), JMV6 in the presence of 50 ng mL^−1^ (JMV6 [ATc 50]), and JMV6 + p*lcpA* in the presence of 50 ng mL^−1^ (JMV6 + p*lcpA* [ATc 50]) strains were performed using a superresolution microscope. Bacteria were stained for DNA (DAPI, blue) and PSII (anti-PSII, green). The merged images show both localizations simultaneously; scale bars, 20 μm. The insets show magnifications of parts of the images; scale bars, 2 μm.

To analyze the localization of PSII at the bacterial surface when its anchoring is impaired due to the limitation of LcpA and LcpB, the conditional-lethal mutant strain JMV6 was cultured with 10 ng mL^−1^ or 50 ng mL^−1^ ATc (Fig. 6). We used the JMV2 strain as a control, which has a second copy of *lcpB* (P*_tet_*-*lcpB* copy at the *ermB* locus). The JMV2 strain has a similar phenotype to the JMV1 strain, confirming that overexpression of *lcpB* due to the induction of the second copy does not affect PSII anchoring and bacterial morphology. In the conditional-lethal mutant strain JMV6, we confirmed that a low induction of *lcpB* (10 ng mL^−1^ ATc) leads to ellipsoid cells. The rod shape was restored in the presence of 50 ng mL^−1^ ATc with or without *lcpA*. Moreover, PSII was still localized at the bacterial surface of the JMV6 strain in the presence of 10 ng mL^−1^ ATc (Fig. 6, JMV6 [ATc 10]). This result was surprising because, according to the previous study of Chu et al. (27), PSII was expected to be found in the supernatant fraction. Our results suggest that after the synthesis of PSII, it is still anchored to its lipid carrier at the plasma membrane, in accordance with previous models (20, 27).

### Part of the surface PSII and Cwp proteins is released in the JMV6 strain.

**FIG 7:**
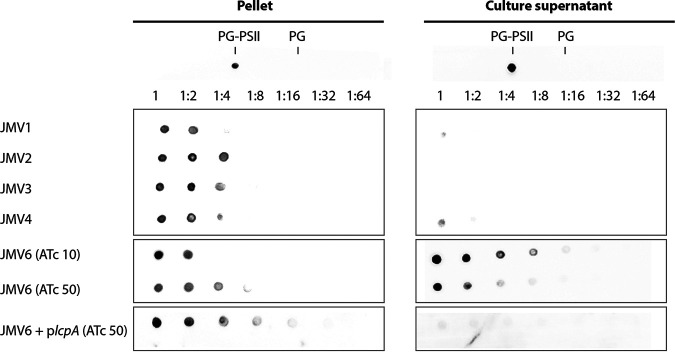
PSII is released into the supernatant of the JMV6 strain in the presence of 10 ng mL^−1^ ATc. Dot blot analysis using specific antibodies targeting PSII was performed on the bacterial surface content (pellet) and supernatant content (culture supernatant) from JMV1, JMV2 JMV3, JMV4, JMV6 grown in the presence of 10 ng mL^−1^ ATc (JMV6 [ATc 10]), JMV6 grown in the presence of 50 ng mL^−1^ ATc (JMV6 [ATc 50]), and JMV6 p*lcpA* grown in the presence of 50 ng mL^−1^ ATc (JMV6 + p*lcpA* [ATc 50]). Each content was diluted up to 1:64. PG-PSII was used as a positive control, and PG was used as a negative control.

To assess the impact of a defect of PSII anchoring to PG, we analyzed the presence of PSII at the bacterial surface and in the supernatant by dot blot analysis (Fig. 7). In the JMV1 parental strain and the JMV2 control strain (P*_tet_*-*lcpB*), PSII was found in the bacterial fraction (pellet), suggesting that it was only associated with the bacterial surface. The same result was observed for the single-*lcp*-mutant strains JMV3 and JMV4. Conversely, in the conditional-lethal *lcp* mutant (JMV6) treated with 10 ng mL^−1^ ATc, PSII was found at the bacterial surface and was released in the culture supernatant. This release of PSII was decreased in the presence of 50 ng mL^−1^ ATc. The phenotype was completely restored in the conditional-lethal mutant strain (JMV6) in the presence of *lcpA* and 50 ng mL^−1^ ATc.

**FIG 8:**
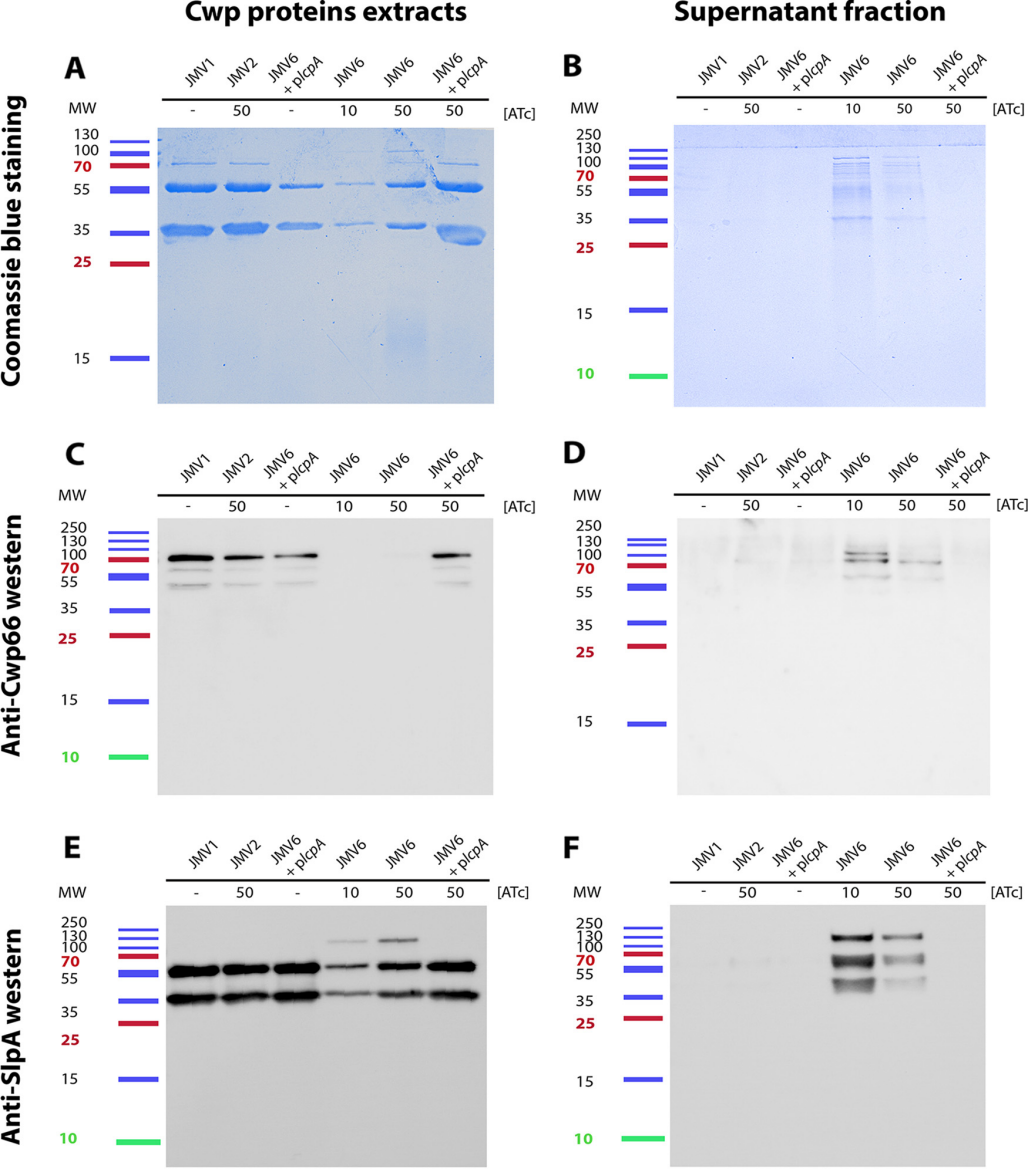
(A to F) PSII anchoring impairment is associated with Cwp proteins released in the culture supernatant. Characterization of surface (A, C, and E) and supernatant (B, D, and F) protein profiles from JMV1, JMV2, JMV6, and JMV6 + p*lcpA* grown in the absence of ATc (−) or in the presence of 10 ng mL^−1^ ATc (10) or in the presence of 50 ng mL^−1^ ATc (50). Coomassie blue staining (A and B), anti-Cwp66 Western blots (C and D), and anti-SlpA Western blots (E and F) were performed. The protein ladder is graduated in kiloDaltons; MW, molecular weight.

**FIG 9:**
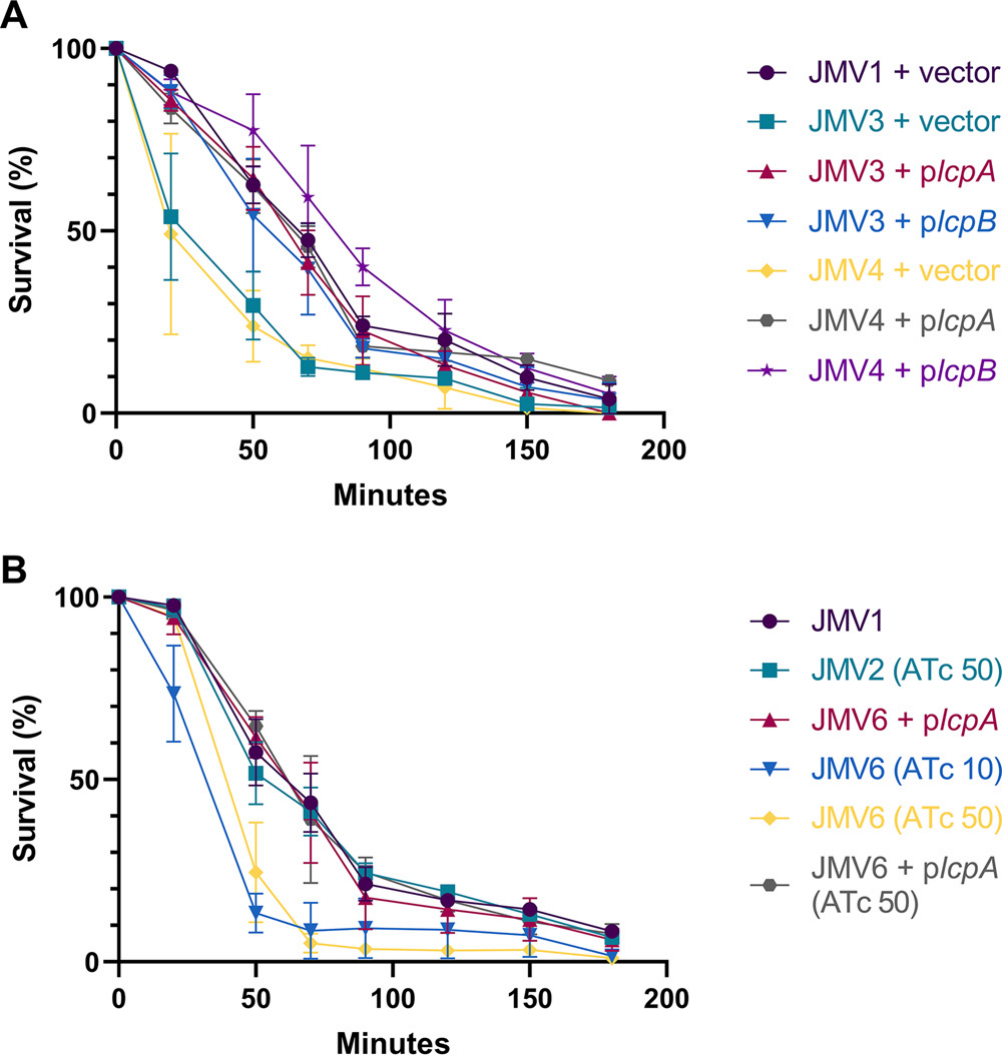
PSII anchoring mutants present an autolysis phenotype. (A) Autolysis of JMV1, JMV3, and JMV4 harboring the empty plasmid pMTL84222 (+ vector), the p*lcpA* plasmid (+ p*lcpA*), or the p*lcpB* plasmid (+ p*lcpB*) were measured. (B) Autolysis of JMV1, JMV2 grown in the presence of 50 ng mL^−1^ ATc (JMV2 [ATc 50]), JMV6 + p*lcpA*, JMV6 grown in the presence of 10 ng mL^−1^ ATc (JMV6 [ATc 10]), JMV6 grown in the presence of 50 ng mL^−1^ ATc (JMV6 [ATc 50]), and JMV6 + p*lcpA* grown in the presence of 50 ng mL^−1^ ATc (JMV6 + p*lcpA* [ATc 50]) was measured. The optical density was measured for 3 h (180 min), and the result is presented as a cell survival percentage. The graph represents the mean of three independent experiments.

Because PSII was released in the supernatant, we assessed whether the Cwp proteins, which are noncovalently linked to PSII, were also found in the supernatant. We showed that the Cwp amount was decreased at the bacterial surface of the conditional-lethal mutant strain (JMV6) in the presence of 10 ng mL^−1^ ATc induction in comparison with the JMV1, JMV2, and JMV6 + *lcpA* strains (Fig. 8A). Cwp proteins of the conditional-lethal mutant strain JMV6 strain in the presence of 10 ng mL^−1^ ATc were found in the supernatant. In comparison, in the presence of 50 ng mL^−1^ ATc, Cwp proteins from the JMV6 strain were more abundant at the bacterial surface. To further characterize which proteins were involved, we performed Western blotting. These analyses allowed us to identify two proteins of the Cwp family, Cwp66 and SlpA, in the supernatant of the conditional-lethal mutant strain JMV6 strain (Fig. 8D and F). Accordingly, Cwp66 was absent from the surface protein extracts (Fig. 8C), and SlpA was found in a lower quantity than in other strains (Fig. 8E). It is noteworthy that SlpA precursor (uncleaved) was found in the conditional-lethal mutant strain (JMV6), suggesting a maturation defect. We analyzed the autolysis profile of all strains to investigate why PSII and the Cwp proteins were found in the supernatant (Fig. 9). We found that the single *lcp* mutants JMV3 and JMV4 autolysed more rapidly than the parental strain (Fig. 9A), and this phenotype was absent when these mutants were complemented with either p*lcpA* or p*lcpB*. The conditional-lethal mutant JMV6 also autolysed more rapidly than the JMV1 strain (Fig. 9B). Again, the impaired phenotype was fully restored in the presence of 50 ng mL^−1^ ATc and *lcpA*. These results suggest that the conditional-lethal mutant strain JMV6 is lysing more rapidly than the parental strain, explaining the partial release of PSII into the culture supernatant.

### Cytoplasmic PG precursors accumulate in response to impaired PSII anchoring to PG.

**FIG 10:**
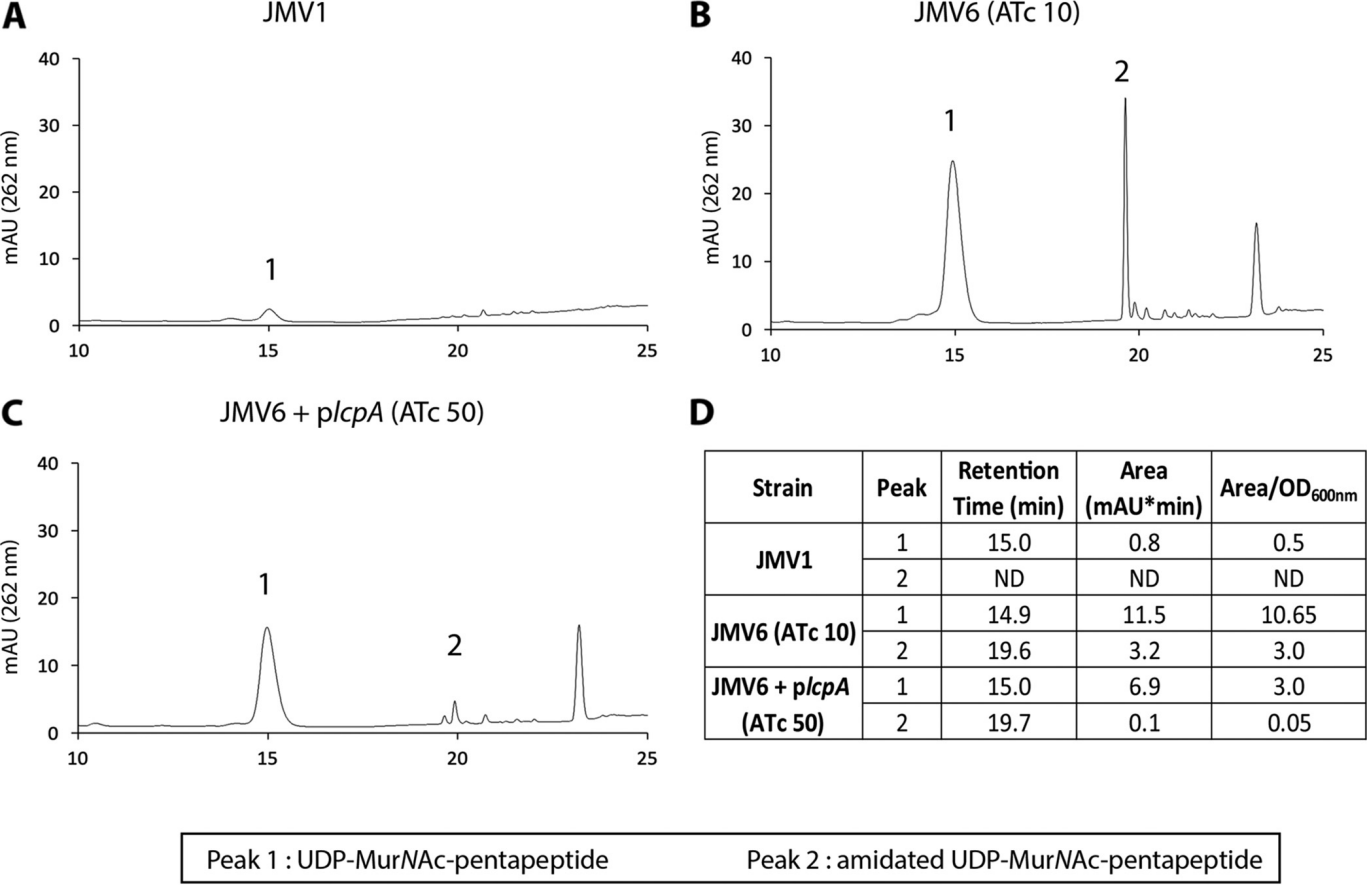
PG cytoplasmic precursors accumulate when PSII anchoring is impaired. (A to C) Purification and quantification of UDP-MurNAc-pentapeptide from JMV1 (A), JMV6 grown in the presence of 10 ng mL^−1^ ATc (JMV6 [ATc 10]) (B), and JMV6 + p*lcpA* grown in the presence of 50 ng mL^−1^ ATc (JMV6 + p*lcpA* [ATc 50]) (C) were performed. (D) Table showing each peak area and area/optical density ratio. In addition, the observed and calculated monoisotopic masses obtained after mass spectrometry analysis are presented in the two last columns; mAU, milli-arbitrary units; Da, Dalton.

Because PSII is attached to the C_55_P carrier during its biosynthesis and until an Lcp protein anchors it to the peptidoglycan, we hypothesized that PSII transfer impairment from the C_55_P carrier to peptidoglycan may limit the availability of this lipid carrier for peptidoglycan synthesis. The extraction of cytoplasmic peptidoglycan precursors was performed for JMV1, conditional-lethal mutant strain JMV6 (10 ng mL^−1^ ATc), and conditional-lethal mutant strain JMV6 + p*lcpA* (50 ng mL^−1^ ATc) (Fig. 10). In the JMV1 strain, only peak 1 was found (Fig. 10A). In the two other tested strains (Fig. 10B and C), peaks 1 and 2 were found. Mass spectrometry analyses (Fig. 10D) indicated that the precursor in peak 1 was UDP-MurNAc-pentapeptide. Analysis of the precursor in peak 2 by tandem mass spectrometry indicated that it differed from UDP-MurNAc-pentapeptide by the amidation of the side chain carboxyl of the diaminopimelyl (DAP) residue located at the third position of the pentapeptide stem. This amidation, attributed to AsnB, was only reported once, when C. difficile was grown in the presence of vancomycin at a sublethal concentration (38). A third peak (Fig. 10B and C) was not identified. UDP-MurNAc-pentapeptide was 21-fold more abundant in JMV6 grown in the presence of 10 ng mL^−1^ ATc than in the parental JMV1 strain. The accumulation of UDP-MurNAc-pentapeptide was less abundant (6-fold instead of 21-fold) in the JMV6 + p*lcpA* strain in the presence of 50 ng mL^−1^ ATc. These results establish that impaired PSII anchoring to peptidoglycan results in the accumulation of the UDP-MurNAc-pentapeptide peptidoglycan precursor. This accumulation is likely to result from a limited availability of the C_55_P lipid carrier for peptidoglycan synthesis due to its sequestration in lipid-linked PSII precursors.

## DISCUSSION

In this study, we characterized LcpA and LcpB as responsible for PSII anchoring to C. difficile PG. In addition, we showed that the activity of these proteins is essential for the viability of C. difficile probably because of an interference with PG synthesis.

In well-studied Gram-positive models like B. subtilis, Streptococcus pneumoniae, and Staphylococcus aureus, *lcp* genes are found in multiple copies in the genome and are at least partially redundant (22–26). Our study confirmed that growth of a *lcpB*-mutant strain is associated with morphological defects, contrary to a *lcpA*-mutant strain (27). Therefore, *lcpB* appears to be more important than *lcpA*, yet *lcpB* is expressed at a lower level than *lcpA*. The morphological and growth defects of the *lcpB* mutant were restored by overexpression of *lcpA*. This overexpression may localize LcpA differently than in the parental strain, allowing complementation by compensating the absence of LcpB at the bacterial surface and suggesting that LcpA and LcpB have partially redundant functions. Similarly, in other bacteria, although redundant in activity, one Lcp has a predominant role, and its absence impacts bacterial physiology more than the others (22, 39, 40). Our immunofluorescence study (Fig. 3) showed that the PSII layer is altered in both single mutants but in different ways, suggesting that these distinct phenotypes can be due to a different localization of the two Lcps at the surface. It is noteworthy that LcpB is predicted to have a transmembrane domain, and LcpA only has a signal peptide domain (https://www.ebi.ac.uk/interpro/), suggesting that LcpB is localized at the membrane and LcpA is secreted. Since PSII is linked to C_55_P at the membrane, a membranous Lcp (LcpB in C. difficile) may be more efficient in transferring it from C_55_P to PG. In contrast, LcpA should be less efficient because of its lack of an N-terminal transmembrane domain, which is atypical among Lcp proteins, since they usually have at least a transmembrane domain (14).

Lcp proteins are phosphotransferases according to Kawai et al. (22) or peptidoglycan-glycopolymer ligases according to Schaefer et al. (40). However, in *lcp* mutants of Staphylococcus aureus and B. subtilis, CWGPs were found to be released (22, 23, 27). There is a discrepancy between these data and the theoretical CWGP synthesis and transfer of the CWGPs from C_55_P to PG. This was explained in Staphylococcus aureus by the activity of CapA1 that catalyzes the cleavage of the pyrophosphate linkage between the CWGP and C_55_P, releasing the CWGP into the supernatant in the absence of Lcp proteins. In contrast, in Streptococcus pneumoniae (26) and our study, we reported that the CWGPs were found both in the supernatant and at the bacterial surface. In our work, it is difficult to know whether this PSII localization is due to the presence of a low level of LcpB (JMV6 in the presence of 10 ng mL^−1^ ATc) or if PSII is still anchored to the C_55_P carrier at the surface. In C. difficile, one gene encodes a putative protein similar to that of CapA1 from Staphylococcus aureus (CD630_11190, 19% identity and 45% similarity), and none were found in the Streptococcus pneumoniae R6 genome. The CD630_11190 putative lipoprotein may have another function than CapA1, but we cannot exclude that the observed release of PSII into the supernatant may be due to this protein together with the observed bacterial lysis (Fig. 9).

Additionally, we showed that PSII release in the conditional-lethal strain was associated with the release of Cwp66 and SlpA surface proteins into the supernatant. Indeed, we were able to detect SlpA at the bacterial surface, which is the most abundant surface protein in C. difficile, but not Cwp66, suggesting that most of the Cwp proteins are not localized at the bacterial surface anymore. In parallel, we observed that SlpA was only partially matured in the JMV6 strain, suggesting that Cwp84 was not efficient in its cleavage. This defect in SlpA cleavage may be due to the Cwp84 localization that was suggested to be first active when positioned at the surface, released after an automaturation, and finally fully active and reassociated with the bacterial surface (41). This last step may be missing due to a probable association with the released PSII instead of the bacterial surface, explaining the partial defect in SlpA cleavage.

Indirectly blocking the recycling of C_55_P (e.g., with cell wall synthesis inhibitors [such as bacitracin and vancomycin]) leads to an accumulation of UDP-MurNAc-pentapeptide in the cytoplasm and bacterial death (42). Because the PSII is predicted to be anchored on the C_55_P lipid carrier during its biosynthesis (3, 27) and until it is transferred by Lcp proteins to the PG (19), we hypothesized that impairment in PSII anchoring could lead to blockage of peptidoglycan biosynthesis through competition between the C_55_P-linked PSII and the synthesis of lipid II that requires free C_55_P. Our results suggest that the sequestration of C_55_P-linked PSII blocks the transfer of UDP-MurNAc-pentapeptide to free C_55_P, leading to its accumulation in the cytoplasm. During this accumulation, the UDP-MurNAc-pentapeptide is amidated (Fig. 10). This amidation of a peptidoglycan precursor was already observed and mediated by AsnB in C. difficile but only in the presence of vancomycin (38). As vancomycin also targets lipid II, we can hypothesize that the accumulation of UDP-MurNAc-pentapeptide may induce the expression of *asnB*, leading to the amidation of peptidoglycan precursors.

UDP-MurNAc-pentapeptide accumulation suggests that PG synthesis is blocked and explains the essentiality of Lcp activity in C. difficile. In B. subtilis, CWGPs are dispensable for cell viability (43), but the absence of the three Lcps is lethal (22). Similarly, in Mycobacterium tuberculosis, Lcp1 (the unique Lcp) was shown to be essential (44). In other bacterial species, this essentiality was not reported, but the absence of Lcp led to defects in growth, morphology, and virulence (23, 40, 45). Our results confirm the importance of the Lcp proteins in bacterial cell wall organization and their essentiality for bacterial physiology and fitness. Since Lcp proteins are mainly found in Gram-positive bacteria and especially in pathogens, they are very good targets for the identification of a new class of antibacterial drugs to counteract the emergence of multidrug-resistant bacteria.

## MATERIALS AND METHODS

### Bacterial strains and growth conditions.

The strains used and constructed in this study are listed in Table 1. All C. difficile strains of this study are isogenic derivatives of the clinical 630 strain (46). C. difficile was grown in brain heart infusion (BHI) medium (BD Difco) at 37°C under anaerobic conditions (Jacomex, 5% H_2_, 5% CO_2_, and 90% N_2_). When needed, BHI was supplemented with 1% defibrinated horse blood, thiamphenicol (Th; 7.5 μg mL^−1^), aztreonam (Az; 16 μg mL^−1^; used to kill parental Escherichia coli during the conjugation process), or erythromycin (Er; 5 μg mL^−1^). Anhydrotetracycline (ATc) was used to induce the P*_tet_* promoter (concentrations from 5 to 250 ng mL^−1^). Growth curves were obtained using a SpectraMax plate reader (Molecular Devices). E. coli was grown aerobically in LB medium at 37°C supplemented, when needed, with ampicillin (Amp; 100 μg mL^−1^), chloramphenicol (Cm; 25 μg mL^−1^), kanamycin (Kn; 40 μg mL^−1^), spectinomycin (Spec; 100 μg mL^−1^), or erythromycin (Er; 150 μg mL^−1^).

**TABLE 1:** Bacterial strains and plasmids used in this study

Name	Genotype or primer sequence	Source or reference
Bacterial strains		
Escherichia coli		
TG1	E. coli K-12: F′ *traD36 lacI*^q^ *lacZ*ΔMIS *proAB*/*supE* Δ(*hdsM*-*mcrB*)	Laboratory stock
HB101 pRK24	E. coli (pRK24): F^−^ Δ(*gpt-proA*)*62 leuB6 glnV44 ara14 galK2 lacY1* Δ(*mcrC-mrr*) *rpsL20* (Srt^R^) *xyl5 mlt1 recA13* pRK24	Laboratory stock
Clostridioides difficile		
630	Clinical strain, Erm^R^	Sebaihia et al. 2006 (46)
630Δ*erm*	Derivative of 630 strain, Erm^S^	Hussain et al. 2005 (37)
JMV1	Derivative of 630 strain, Erm^S^, Δ(*CD630_20100*, *CD630_20091*, *CD630_20090*, *CD630_20080*, *CD630_20071*, *CD630_20070*)	This work
JMV2 (630 P*_tet_-lcpB)*	Derivative of 630 strain, Erm^S^, Δ(*CD630_20100*, *CD630_20091*, *CD630_20090*, *CD630_20080*, *CD630_20071*, *CD630_20070*)::P*_tet_*-*lcpB*	This work
JMV3 (Δ*lcpA)*	JMV1 Δ*lcpA*	This work
JMV4	JMV1 Δ*lcpB*	This work
JMV5 (Δ*lcpB* P*_tet_-lcpB)*	JMV2 Δ*lcpB*	This work
JMV6 (Δ*lcpA* Δ*lcpB* P*_tet_*-*lcpB*)	JMV2 Δ*lcpA* Δ*lcpB*	This work
Plasmids and vectors		
pMSR	Circular cloning vector, 5,624 nucleotides, *catP*, α*lacZ,* P*_tet_*-*CD2517* (toxin), pseudosuicide plasmid, Cm^R^	Gift from J. Peltier (36)
pBLUNT	Linear cloning vector from Invitrogen, Kn^R^	Invitrogen
pAT28	Mobilizable shuttle plasmid, Spc^R^	Trieu-Cuot et al. 1990 (57)
pRPF185	P*_tet_-gusA* Tm^R^, expression and cloning vector	Fagan et al. 2011 (58)
pMTL-83151	Cm^R^ cloning vector, pCB102 replicative origin	Heap et al. 2009 (59)
pMTL-84151	Cm^R^ cloning vector, pCD6 replicative origin	
pMTL-84222	Erm^R^ cloning vector, pCD6 replicative origin	
pJV4	pBLUNTΩ*aadA*, spectinomycin resistance gene flanked by BsaI sites, Kn^R^	This work
pJV5	pMSR derivative, used to construct pJV8, Cm^R^, and Sp^R^	This work
pJV6	pMTL-83151Δ*catP*Ω*ermB*, Erm^R^	This work
pJV7	pMSRΩ*aadA,* spectinomycin resistance gene flanked by BsaI sites, Cm^R^, and Sp^R^	This work
pJV8	pMSR derivative, plasmid used for *erm* locus deletion, Cm^R^, and Sp^R^	This work
pJV10	pJV7Δ*catP*Ω*ermB*, Erm^R^, and Sp^R^	This work
pJV11	pJV10 derivative, plasmid used for *lcpA* deletion, Erm^R^	This work
pJV12	pJV10 derivative, plasmid used for *lcpB* deletion, Erm^R^	This work
pJV13	pJV10 derivative, plasmid used for *lcp* region deletion, Erm^R^	This work
pTC131	pMTL-84151Ω*aadA,* spectinomycin resistance gene flanked by BsaI sites, Cm^R^, and Sp^R^	This work
pMEZ5	pTC131Δ*catP*Ω*ermB,* Erm^R^, and Sp^R^	This work
p*lcpA* (pMEZ12)	pMEZ-5ΩP*_lcpA_-lcpA*, Erm^R^	This work
pJV20	pTC131ΩP*_lcpB_-lcpB*, Cm^R^	This work
p*lcpB* (pJV21)	pMTL-84222ΩP*_lcpB_-lcpB*, Erm^R^	This work
pJV27	pJV8ΩP*_tet_*-*lcpB*, Cm^R^	This work
pMDR1	pTC131Ω*aphA*Ω*gusA*, Cm^R^, and Kn^R^	This work
pMDR2	pMDR1Δ*aphA*Ω*aadA*, Cm^R^, and Sp^R^	This work
pMDR5	pMDR2Δ*aadAΩ*P*_lcpB_*, Cm^R^	This work
pMDR8	pMDR2Δ*aadAΩ*P*_lcpA_*, Cm^R^	This work

### Molecular biology.

Plasmid extractions, gel extractions, and PCR purifications were performed using the Omega E.Z.N.A plasmid DNA minikit, gel extraction kit, and cycle pure kit, according to the manufacturer’s instructions. PCRs were performed using high-fidelity Phusion DNA polymerase for gene amplification on genomic DNA and mutant screening of C. difficile. In contrast, the *Taq* DNA polymerase was used for screening steps in E. coli.

### Construction of plasmids.

A list of plasmids and primers used in this study can be found in Table 1 (plasmids) and Tables S1 and S2 in the supplemental material (primers). The construction of all plasmids is detailed in Text S1. The plasmids used in this study were constructed using either the Gibson assembly protocol from New England BioLabs (NEB) (47) or Golden Gate assembly from NEB (48, 49) cloning techniques. For Golden Gate assembly, the primers were designed using the NEB Builder assembly tool.

### Mutant strain construction.

Plasmids were transferred from E. coli HB101 (pRK24) to C. difficile via heterogramic conjugation (between E. coli and C. difficile), following the previously described protocol (50). The single- and double-crossover events were screened based on the pseudosuicide plasmid pMSR following the appropriate protocol described by Peltier (36), with some modifications. As we replaced the ORFs with a *catP* gene, a first quick screen for the second crossover event was performed by restreaking clones on BHI supplemented with a thiamphenicol agar plate. Then, only Th^R^ clones were checked by PCR using appropriate primers (Table S2).

### Construction of JMV1, JMV3, and JMV4 strains.

The JMV1 strain is an Δ*ermB* region derivative of the clinical 630 strain. The deletion was made by replacing the complete *ermB* region (genes *CD630_20100* [*ermB*], *CD630_20091*, *CD630_20090*, *CD630_20080*, *CD630_20071*, and *CD630_20070* [*ermB*]) with a spectinomycin resistance gene. This replacement was made by an allelic exchange technique (36) using the pJV8 plasmid.

The single *lcp* mutants JMV3 and JMV4 are derivatives of the JMV1 strain, where the ORF was replaced with a thiamphenicol resistance gene. The deletion of *CD630_27650* (*lcpA*) and *CD630_27660* (*lcpB*) was made by allelic exchange using deletion plasmids pJV11 and pJV12, respectively. The mutants were PCR verified using the primer pairs JV85/JV90 and JV86/JV91 for the JMV3 mutant and JV88/JV90 and JV87/JV91 for the JMV4 strain (Table S2).

### Conditional-lethal mutant construction.

The insertion of the P*_tet_-lcpB* into the *erm* locus was made using the pJV27 plasmid, and the resulting strain JMV2 was PCR verified using the JV99/JV100 primers. The deletion of both *lcp* genes was then made using the pJV13 plasmid and the use of 100 ng mL^−1^ ATc, giving rise to the conditional-lethal mutant strain JMV6, which can be PCR checked using primers JV85/JV90 and JV91/JV87 (Table S2).

### β-Glucuronidase assay.

The β-glucuronidase assay was performed as described in Ammam et al. (38).

### Growth and autolysis curves.

Growth and autolysis curves were performed using a SpectraMax plate reader. To ensure anaerobic conditions, 96-well plates were covered with an adhesive film in the anaerobic chamber. The cultures were grown in BHI at an approximate optical density of 0.1 from overnight preculture of different strains. Growth and autolysis curves were performed at 37°C.

### Cwp proteins and supernatant proteins extractions.

Cwp proteins were isolated from intact C. difficile bacteria using low-pH glycine, as described previously by Fagan et al. (51). The optical density was systematically adjusted to 1 for all strains before protein extraction. Supernatant protein fractions were obtained by harvesting bacteria (20,000 × *g*, 15 min, 4°C) from overnight cultures previously adjusted to an optical density (600 nm) of 1 and then precipitated with 10% trichloroacetic acid (on ice, 4 h). The pellet was finally resuspended in Tris 50 mM, pH 7.4.

### Preparation of antigens and antibodies against PSII and SlpA.

Surface polysaccharide II was isolated using the protocol described by Cox (52). The detection of glycopolymers in FPLC (fast protein liquid chromatography) fractions was accomplished by the phenol-sulfuric assay (53). The fractions of interest were freeze dried and analyzed by ^1^H and ^31^P NMR (ENS Lyon) to confirm PSII purification. Purified NMR-confirmed PSII was then conjugated to bovine serum albumin (BSA). The coupling reaction proceeded according to the protocol described by Romano (54), with cyanoborohydride (NaBH_3_CN) as a coupling agent. The resulting glycoconjugate antigen (PSII-BSA) was submitted to Covalab (France) for rabbit immunization (four injections with 50 μg of the glycoconjugate per animal). Specificity of the purified PSII was confirmed by dot blot and NMR-confirmed peptidoglycan (PG) and PG-PSII extracts.

SlpA was purified as described by Bruxelle et al. (55) and was submitted to Covalab (France) for guinea pig immunization (four injections with 22.5 μg of the protein per animal). Specificity of the polyclonal antibodies was performed by Western blotting.

### Immunodetection.

For Western blotting, the following antibodies were used: anti-SlpA antibodies (guinea pig) diluted at 1:5,000 and anti-Cwp66 antibodies (rabbit) diluted at 1:10,000. For the dot blot analysis, anti-PSII antibodies (rabbit) diluted 1:10,000 were used. Antibody binding was revealed with anti-rabbit Immobilon Western chemiluminescent horseradish peroxidase (HRP) substrate (Merck), and imaging was performed on a Fusion Fx imaging system (Vilber Lourmat).

### PSII visualization by superresolution confocal microscopy.

A 16-h culture was diluted to obtain 10^8^ cells mL^−1^ using a Kovaslide system, and 20 μL of this diluted culture was deposited on a thin, round coverslip. After drying, the slides were stained in Trisbuffered saline (TBS)-Tween and 5% BSA, washed, and incubated with the primary antibody (anti-PSII, 1:200) for 1 h, incubated with the secondary antibody (StarRED from Abberior, 1:500) for 1 h, and incubated with Hoechst (1:2,000) to visualize DNA. Washes were performed between each step. Finally, the coverslip was mounted on a slide with mounting medium (Abberior Mount Solid) and stored overnight at 4°C before imaging on a STEDYCON superresolution microscope (Abberior).

### PG cytoplasmic precursor extraction and analysis.

The protocol described previously by Cremniter et al. (56) was used with some modifications. Bacteria were grown in 500 mL of brain heart infusion broth overnight and subjected to ice-cold formic acid (47 mL, 1.1 M) extraction for 30 min at 4°C without prior bacitracin treatment.

To allow comparison of the different strains whose culture optical densities were not equal, we calculated the ratio peak area/optical density, presented in the results. The extract was centrifuged (7,000 × *g* for 15 min at 4°C), and the supernatant was loaded into a gel filtration column (Sephadex G-25) for desalting. The fraction of elution was lyophilized and resuspended in 10 mL of water. One hundred microliters of this cytoplasmic precursor solution was loaded onto a reverse-phase high-performance liquid chromatography (RP-HPLC) machine in a C_18_ column (Hypersil GOLD aQ; 250 × 4.6 mm, 3 μm; Thermo Scientific) at a flow rate of 1 mL/min. A linear gradient (0% to 20%) was applied between 13 and 33 min at 25°C (buffer A, 50 mM ammonium formate (pH 4.4); buffer B, 100% methanol). Absorbance was monitored at 262 nm, and the peak corresponding to the major cytoplasmic precursor was collected, lyophilized, and resuspended in 20 μL of water. Ten microliters was analyzed by mass spectrometry on a Bruker Daltonics maXis high-resolution mass spectrometer (Bremen, Germany) operating in the positive mode (analytical platform of the Muséum National d’Histoire Naturelle, Paris, France). Mass spectral data were analyzed using Bruker Compass DataAnalysis 4.3.

### Statistics.

Statistical analyses were conducted using GraphPad Prism (version 9.0.0, GraphPad Software, San Diego, CA, USA; www.graphpad.com). The *P* value is indicated for all comparisons when differences are statistically significant.

### Data availability.

The data for this study can be obtained from the corresponding author.
