# Supplementary material for: Polysaccharide II Surface Anchoring, the Achilles’ Heel of Clostridioides difficile
Source: Microbiol Spectr. 2023 Feb 23;11(2):e04227-22. doi: 10.1128/spectrum.04227-22 (PMC10100865; doi:10.1128/spectrum.04227-22)
Supplement: Supplemental file 1 — Supplemental material. Download spectrum.04227-22-s0001.pdf, PDF file, 1.0 MB [file spectrum.04227-22-s0001.pdf]

# SUPPLEMENTARY DATA

## Tables

Table S1: Primers used for plasmid constructions

| Name | Use: Construction of … | 5’ Primer tail | Primer |
|---|---|---|---|
| JV54 | pJV4 | GGCTACTGCCAGAGACC | GGAAAAGATCCGGGGGATCGATCCTCTAG |
| JV55 | | GGCTACTTGCTGAGACC | TTAGCCTAATTGAGAGAAGTTTCTATAG |
| JV50 | pJV5 | GCAGATAAATAA | TGCCAGAGACCGGAAAAGATCCGGG |
| JV51 | | GTGTAACTTTCC | TTGCTGAGACCTTAGCCTAATTGAGAGAAG |
| JV52 | | AAGGTCTCAGCAA | GGAAAGTTACACGTTACTAAAGGGAATG |
| JV53 | | AGCTTGCATGTCTGCAGGCCT CGAG | CTTGTCGGTAGCTGTGGTATGGATTG |
| TC287 | pJV6 | | GTTTAAACTCCTTTTTGATAATCTC |
| TC288 | | | CGCTTATAATCCATAACAATCATCC |
| TC289 | | TATGGATTATAAGC | GCCGAAGCAAACTTAAGAGTGTG |
| TC290 | | AAAAGGAGTTTAAAC | AAACACATTCCCTTTAGTAACGTG |
| JV48 | pJV8 | AGATTGTAGTTCTTCGGATCCTCTA | GACTATGGAACGTACACTTTTGGCG |
| JV49 | | CCGGTCTCTGGCA | TTATTTATCTGCGTAATCACTGTTTTTAGTC |
| JV58 | pJV11 (*lcpA* deletion plasmid) | CGATAGGGTCTCGTTGC | GTCACCAAATACCATAGTTTCTT |
| JV59 | | CGATAGGGTCTCG | CATTAATATCCCCTACTTTCTAAATTTTTTAAT |
| JV60 | | CGATAGGGTCTCC | AATGGTATTTGAAAAAATTGATAAAAATAGT |
| JV61 | | CGATAGGGTCTCC | TAACTATTTATCAATTCCTGCAATTC |
| JV62 | | CGATAGGGTCTCG | GTTAAAAATTCCAAAACAAACCAATAATTTG |
| JV63 | | CGATAGGGTCTCG | TGCCTTAAGTCGCCCATTTTTAAAAC |
| JV64 | pJV12 (*lcpB* deletion plasmid) | AGTACCGGTCTC | CTTGCCTATTGATAATAAAAATAAAAGTCTTAAGC T |
| JV65 | | AGTACCGGTCTC | CCATAAGTACCCCTTCTTTCTTCTT |
| JV66 | | AGTACCGGTCTCC | TATGGTATTTGAAAAAATTGATAAAAATAGT |
| JV67 | | AGTACCGGTCTCC | TAACTATTTATCAATTCCTGCAATTC |
| JV68 | | AGTACCGGTCTCC | GTTAAAAAATTCAACATAAAGTTTATTAAAAAGTA TAAGA |
| JV69 | | AGTACCGGTCTC | CTGCCTTGATGGTATAACATCAACACC |
| JV70 | pJV13 (*lcpAB* deletion plasmid) | TTCCTGGGTCTCCCC | TAATATCCCCTACTTTCTAAATTTTTTAAT |
| JV71 | | TTCCTGGGTCTCC | TAGGCCGGCCAAGTGGGCAA |
| JV72 | | TTCCTGGGTCTCCTTCT | TAGGGTAACAAAAAACACCGTATTTCTACGATGT |
| JV73 | | TTCCTGGGTCTCC | AGAAAATTCAACATAAAGTTTATTAAAAAGTATAA GATTAATTACT |
| TC381 | pMEZ5 | | CTTTTTGATAATCTCATGACC |
| TC382 | | | GAAATGCAAGTTTCTAACTAAC |
| TC383 | | | TAGTTAGAAACTTGCATTTCACTTGCAT TTCGGCCGGCCGAAGC |
| TC384 | | | GTCATGAGATTATCAAAAAGACACATT CCCTTTAGTAACGTGTAACTTTC |
| TC403 | | GGCTACGGTCTCTTTGC | ACATTTCCTCCCCCAAATTATTAATTTATAAT TATTTTTTATTAATTTTTATC |

| | | | |
|---|---|---|---|
| TC404 | pMEZ12 (p*lcpA*) | GGCTACGGTCTCTTGCC | CTAATCTTCAACCATAATATCTTTAAATATGAAATC |
| JV101 | pJV21 (p*lcpB*) | GGCTACGGTCTCA | TTGCTTTCTACTGAAAATGGTAGAAAAATAG |
| JV102 | | GGCTACGGTCTC | CTGCCTTATTGTTTAAACTCTATGTCATTAAATATAAAATC |
| JV136 | pJV27 (insertion of $P_{tet}$-*lcpB* in the chromosome) | GGCTACGGTCTCTTGCC | TAAAAATAAGAAGCCTGCATTTGC |
| JV137 | | GGCTACGGTCTCT | TCCTTTACTGCAGGAGCTC |
| JV138 | | GGCTACGGTCTCTAGGAGAAAATT | TTTTGTCAAAATTAAAGAAATTTGTTATAC |
| JV139 | | GGCTACGGTCTCCTTGC | TTATTGTTTAAACTCTATGTCATTAAATATAAAATC |

Table S2: primers used for PCR check of mutants

| Name | Use : PCR check of … | Primer |
|---|---|---|
| JV85 | *lcp* ORF replacement by *catP* (JMV3, JMV4, and JMV6) | GCACTTTTCATCATTTCCACATCATTTAAC |
| JV86 | | GAATTTCATCATCAATAGGAAATTCAAATTGC |
| JV87 | | CAAATTCAGATACAGTAGTATTAGTAAATG |
| JV88 | | CTATACAAGATGATAGTATAAATACAGAGGC |
| JV90 | | GTACAAGGTACACTTGCAAAGTAGTGGTC |
| JV91 | | CAAGTTCATCACGCAGTATGTGACGG |
| JV99 | $P_{tet}$-*lcpB* insertion in *erm* locus (JMV2 and JMV6) | GGCATGGCACATCAGTAAAAATTGAATAC |
| JV100 | | CGGTCACGGTGTAATCTTCTGTGACTGCC |
| TC153 | | GAATATTACTACCAAGAAAGCCAGTAG |
| TC154 | | GACATATTACACGATTTTATATTTAATGAC |

# Figures

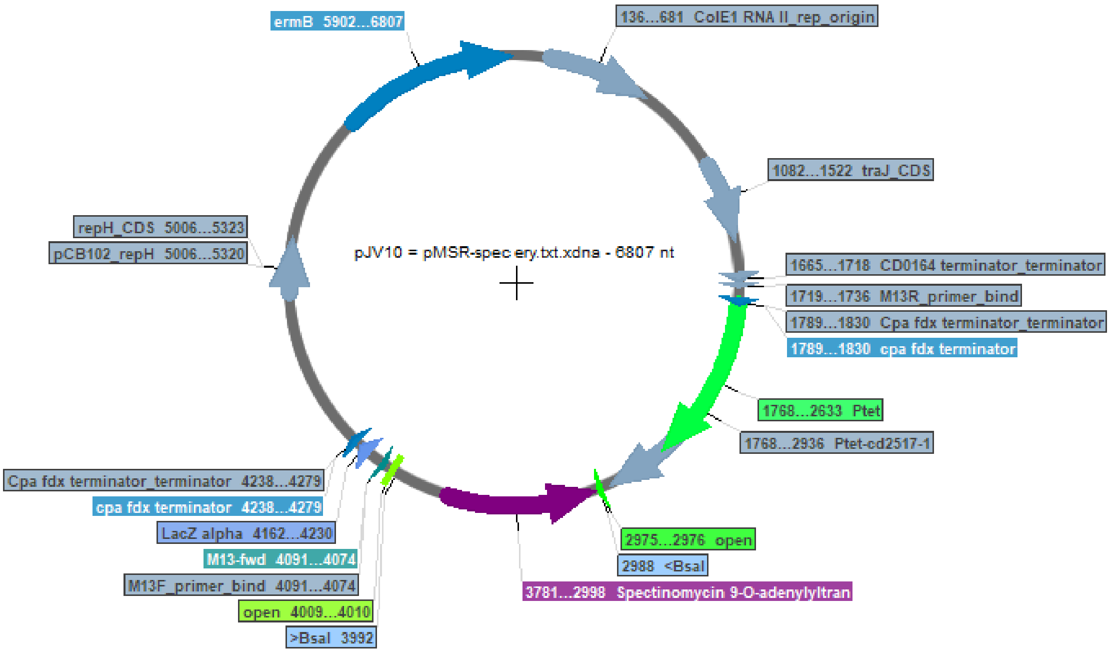


Figure S1

**Graphic map of the pJV10 plasmid used to construct deletion plasmids of the *lcp***

On this graphic map of the pJV10 plasmid, created by Serial Cloner, the spectinomycin resistance gene flanked by BsaI sites to allow Golden Gate assembly, an erythromycin resistance gene, and the $P_{tet}$-CD2517 (Toxin) from the pMSR to facilitate counterselection during the allelic exchange are shown.

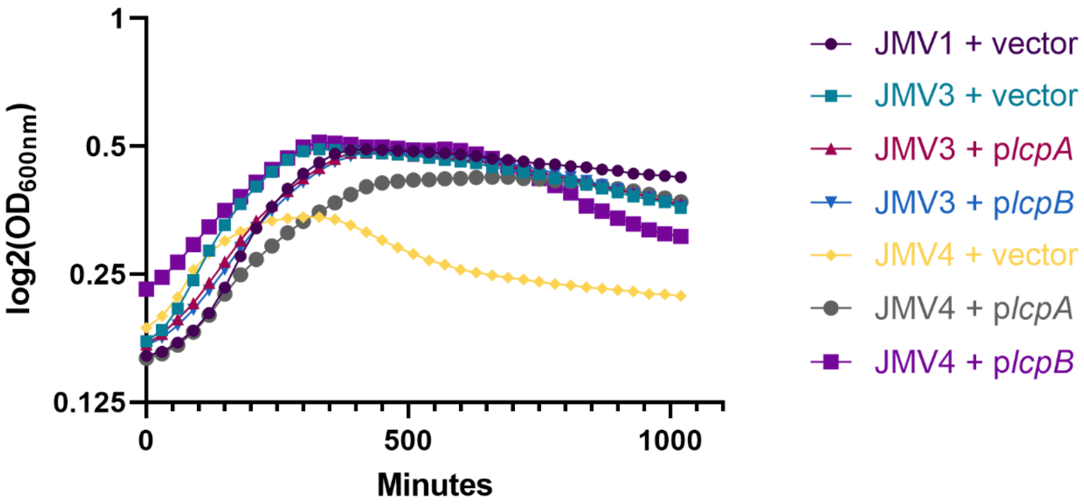


Figure S2

**The Δ*lcpB* strain presents an altered growth**

Growth curve of single mutant strains of *lcpA* (JMV3) *and lcpB* (JMV4), harboring either the pMTL84222, or the p*lcpA* or p*lcpB* plasmid. The growth was observed in BHI medium for 17 hours (1020 minutes). The graph represents the mean of 3 independent experiments.

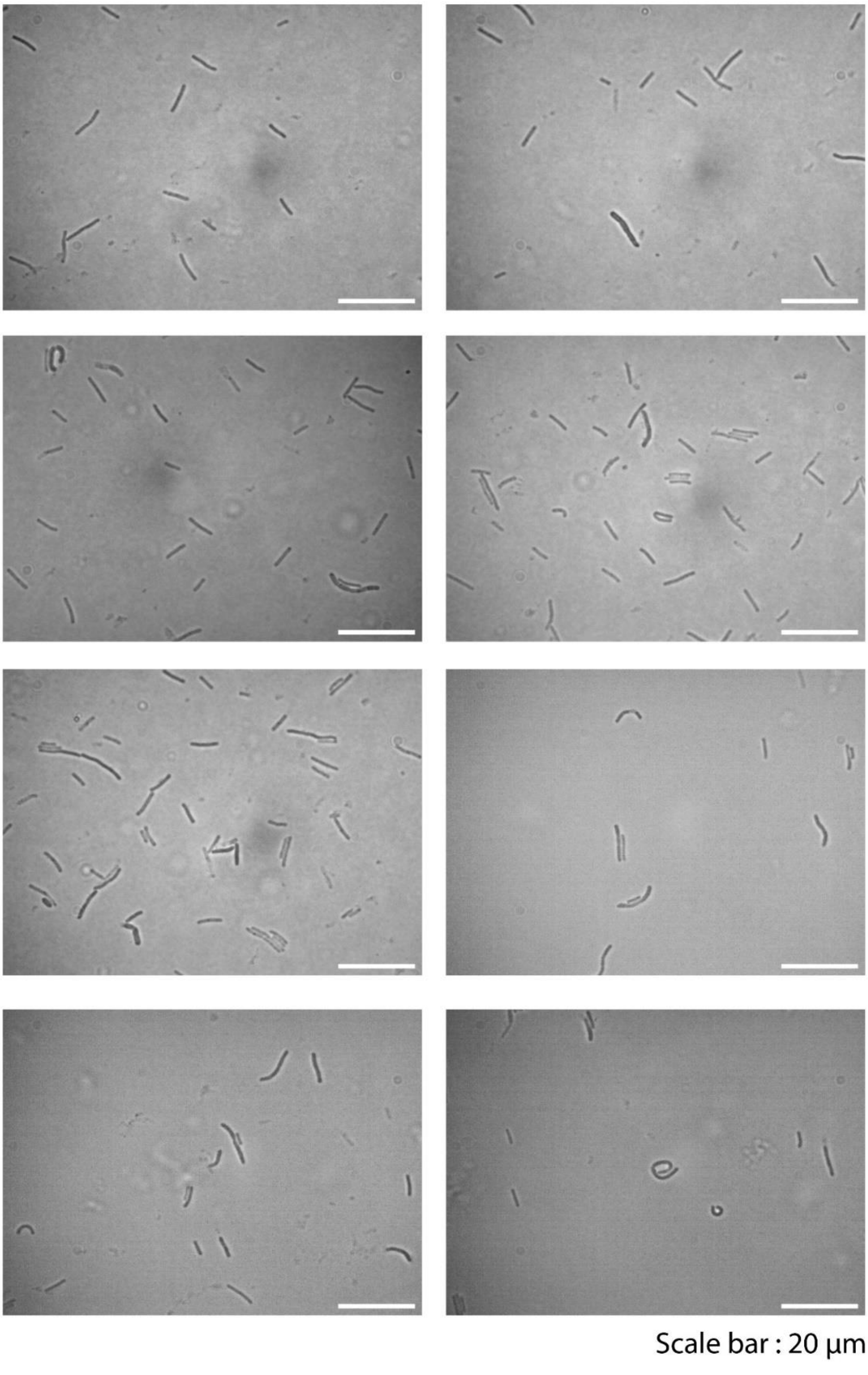


Figure S3

**The Δ*lcpB* mutant (JMV4) is thicker, curved, or inflated in liquid culture.**

These panels present additional pictures of the JMV4 strain observed in optic microscopy. The scale bar represents 20 µm.

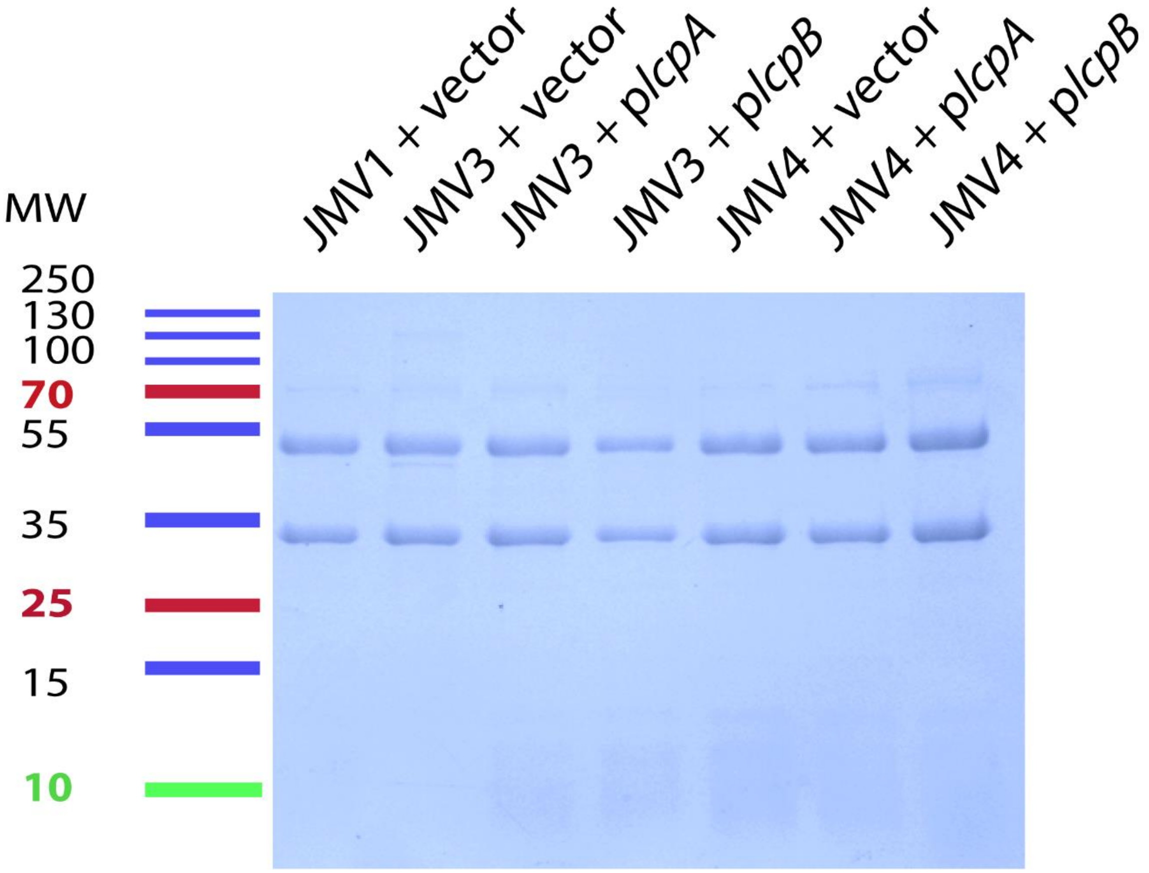


Figure S4

**The single *lcp* mutants JMV3 and JMV4 exhibit a normal S-layer content**

This Coomassie staining of Cwp protein extractions shows that the Cwp content of the S-layer of JMV1, JMV3 and JMV4 harboring either pMTL84222 (vector), p*lcpA* or p*lcpB* plasmid. The protein ladder is graduated in kg Dalton (kDa). MW: molecular weight.

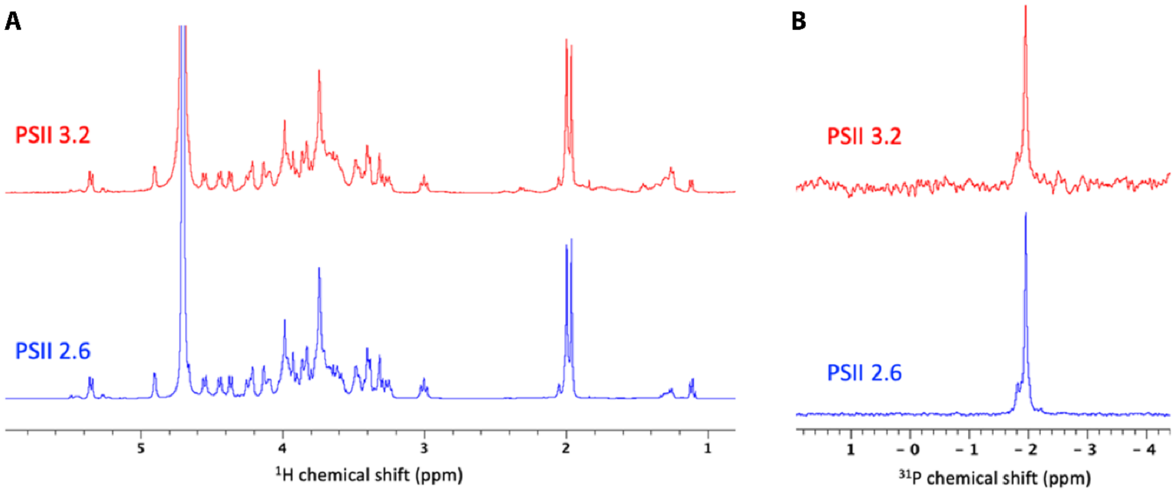


Figure S5

**The PSII was obtained and the absence of contamination with LTA was confirmed by NMR.**

$^{1}$H (**A**) and $^{31}$P (**B**) NMR spectra of the PSII extracted from culture pellets of the 630 strain. Two samples were sent for analysis, named PSII 3.2 and PSII 2.6. Both were confirmed to contain PSII. The chemical shift is measured in part-per-million (ppm).

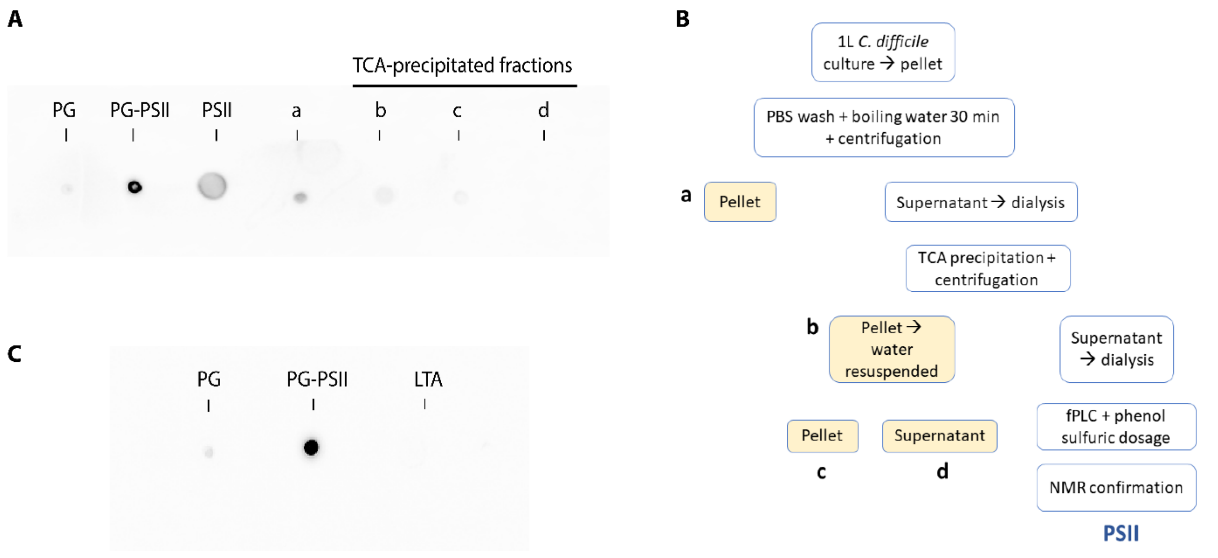


Figure S6

**The immunization led to antibodies production and these antibodies showed good specificity for the PSII**

This dot blot assay shows that the antibodies produced by the rabbits recognize well the RMN-verified PSII (**A**) of *C. difficile* and the PG linked PSII (PG-PSII) (**A** and **C**) and do not cross-react with peptidoglycan (**A** and **C**) or lipoteichoic acid (LTA) (**C**) of *C. difficile*. Moreover, different samples at different stages of the purification process were tested (**B**). Briefly, PSII purification protocol was performed as followed (white boxes, steps of PSII purification, yellow boxes, potential contaminant molecules) : 1 litter of *C. difficile* culture was pelleted. Pellet was washed in PBS and boiled in water for 30 minutes. After centrifugation, pellet (a) was tested to know whether some PSII were not recovered. The supernatant was further used for purification and a TCA precipitation was performed. After centrifugation, pellet (b) was tested to know whether some PSII was not recovered; it was resuspended in water and centrifuged again, giving pellet (c). The supernatant (d) was dialyzed and applied on fPLC. PSII was recovered, dosage was performed by phenol sulfuric method and PSII was analyzed by NMR.

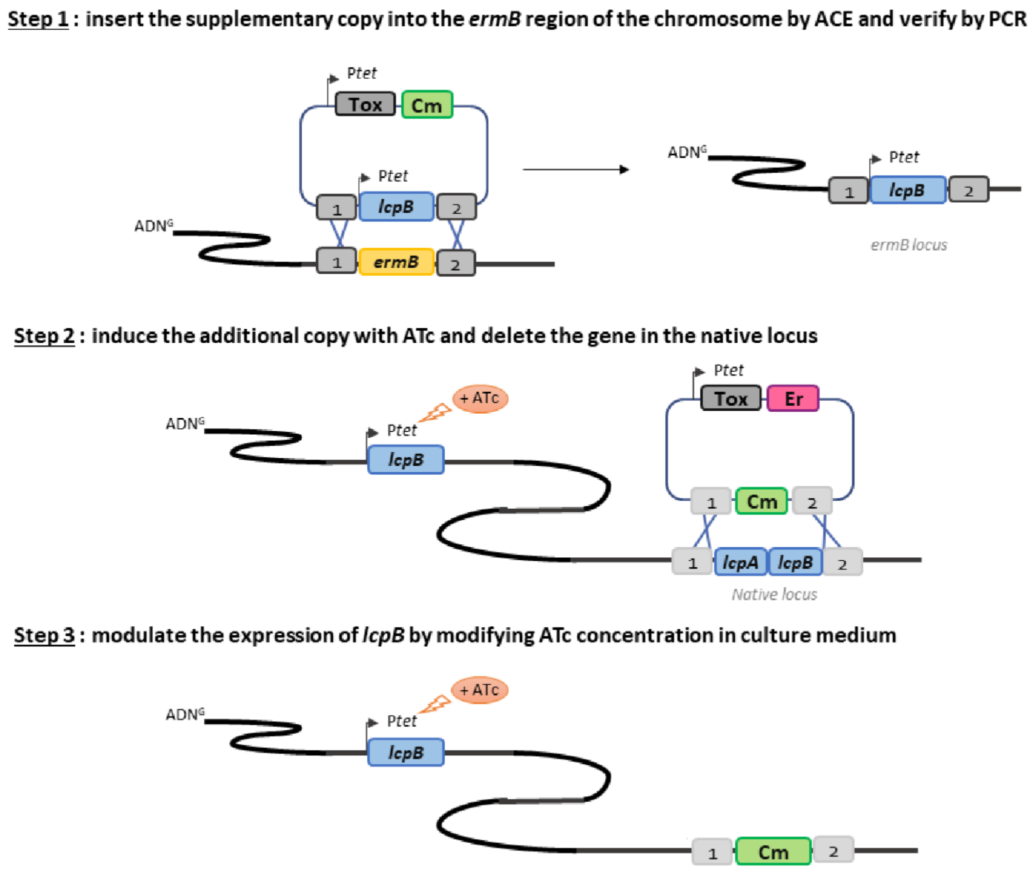


Figure S7

**A new strategy designed to construct a conditional-lethal mutant in *C. difficile***

Schematic representation of the strategy used to create a conditional-lethal mutant of *C. difficile lcpA* and *lcpB* genes. The strategy consists in three major steps: first the insertion of an inducible copy of *lcpB* in the *erm* locus of the chromosome (under control of $P_{tet}$), then the deletion of both *lcpA* and *lcpB* by replacing the ORFs with a *catP* gene, and finally the control of the *l* expression of *lcpB* thanks to ATc induction.

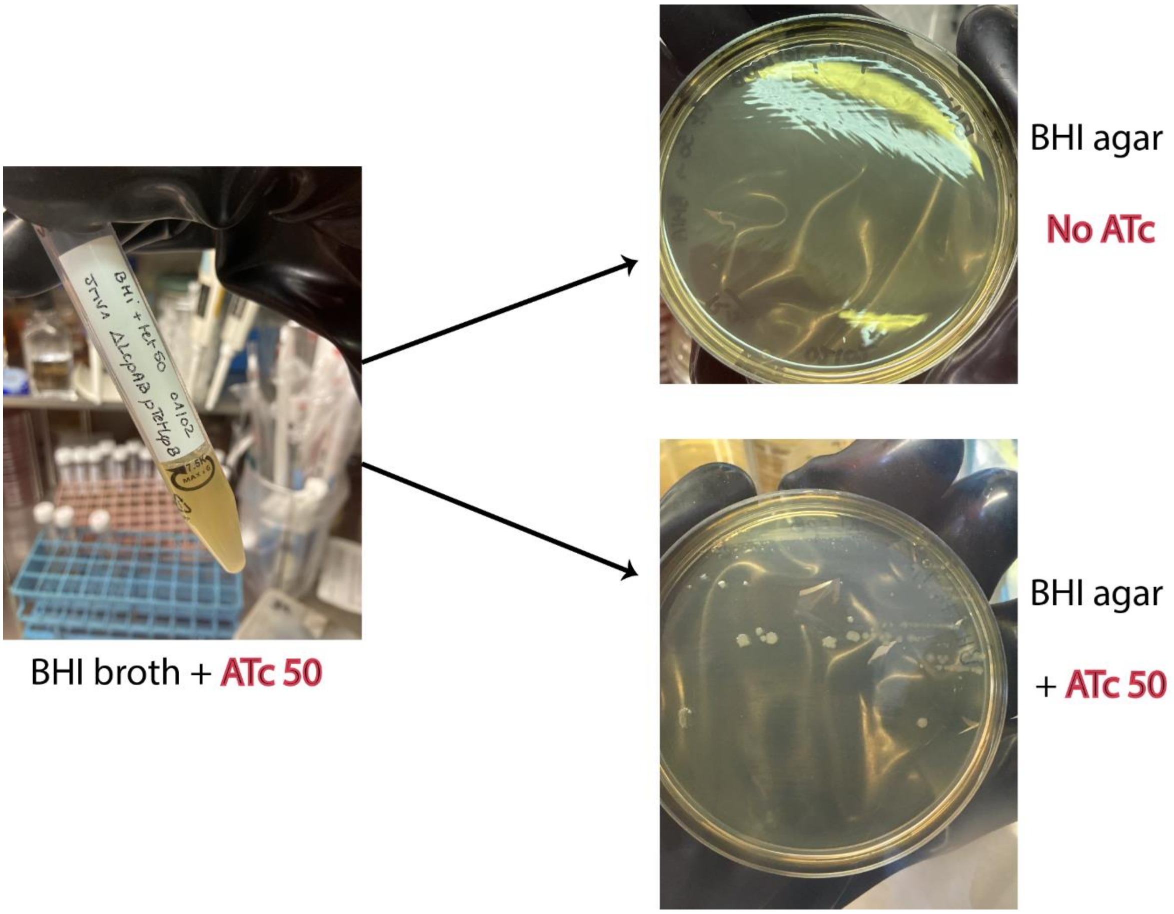


Figure S8

**The anchoring of PSII is essential for *C. difficile* growth**

The conditional-lethal mutant pre-cultured overnight in liquid BHI in the presence of 50 ng.mL$^{-1}$ATc is not able to grow on a BHI plate without ATc but grows correctly in the presence of ATc at 50 ng.mL$^{-1}$.

# Texts

## Text S1: construction of plasmids for the study

The plasmids used in this study were constructed using either the Gibson assembly protocol from NEB (23) or the Golden Gate assembly from NEB (24, 25) cloning technique. For Golden Gate assembly, the primers were designed using the NEB Builder® assembly tool.

### Construction of deletion plasmid for the *ermB* locus

pJV4: the spectinomycin cassette was amplified from pAT28 and flanked with BsaI sites using JV54/JV55 primers. The PCR product was inserted in the pBLUNT cloning vector by blunt-end DNA cloning to give pJV4.

pJV5: the spectinomycin cassette flanked with BsaI sites was amplified from pAT28 using JV50/JV51 primers, and the downstream region of *ermB* locus was amplified from genomic DNA of 630 strain using JV52/53 primers. Both PCR products were assembled using the Gibson assembly protocol (NEB Biolabs) to give pJV5.

pJV7: the spectinomycin cassette flanked with BsaI sites was extracted from pJV4 using restriction digestion with XhoI and BamHI. The pMSR was opened by restriction digestion with XhoI and BamHI. The spectinomycin cassette was then cloned into the linearized pMSR plasmid by a classical ligation process.

pJV8: the upstream region of the *ermB* locus was amplified from genomic DNA of 630 strain using JV48/JV49 primers, and the spectinomycin + downstream region of *ermB* locus fragment was amplified from pJV5 using JV50/JV53 primers, and the pMSR plasmid was amplified using JV46/JV47 primers. The three PCR products were assembled using the Gibson assembly protocol (NEB Biolabs) to give pJV8.

## Constructing deletion plasmids for *lcpA*, *lcpB* and the conditional-lethal deletion of both *lcp*

pJV6: the *ermB* cassette was amplified from pMTL84222 using TC289/TC290 primers, and pMTL83151 was amplified using TC287/TC288 primers. Both PCR products were assembled using the Gibson assembly protocol (NEB Biolabs) to give pJV6. pJV10: this plasmid results from the subcloning of pJV7 (fragment with the spectinomycin resistance cassette flanked by BsaI sites and $P_{tet}$-CD2517 toxin) into pJV6 ($Erm^R$) using the restriction enzymes SacII and XhoI.

pJV11: the upstream and downstream regions of *lcpA* were amplified from genomic DNA of 630 strain using respectively JV58/59 and JV62/JV63 primers. The *catP* cassette was amplified from the pMSR plasmid using JV60/JV61 primers. The three PCR products were inserted in the pJV10 using the Golden Gate assembly protocol (NEB Biolabs) to give pJV11.

pJV12: the upstream and downstreamregions of *lcpB* were amplified from genomic DNA of 630 strain using respectively JV64/65 and JV68/JV69 primers. The *catP* cassette was amplified from the pMSR plasmid using JV66/JV67 primers. The three PCR products were inserted in the pJV10 using the Golden Gate assembly protocol (NEB Biolabs) to give pJV11.

pJV13: the upstream region of *lcpA* and the downstream region of *lcpB* were amplified from genomic DNA of 630 strain using respectively JV58/JV70 and JV73/JV69 primers. The *catP* cassette was amplified with its promoter from the pMSR plasmid using JV71/JV72 primers. The three PCR products were inserted in the pJV10 using the Golden Gate assembly protocol (NEB Biolabs) to give pJV11.

## Constructing complementation plasmids for *lcpA* and *lcpB*

pTC131: pJV4 was digested using BamHI/XhoI restriction enzymes, and the DNA fragment Spec of approximately 1kb was ligated pMTL84151 previously digested by BamHI/XhoI restriction enzymes.

pMEZ5: The *ermB* cassette from pMTL-84222 was amplified using TC383/TC384 primers, and pTC131 was amplified using TC381 and TC382 primers. Both PCR products were assembled using the Gibson assembly protocol (NEB Biolabs) to give pMEZ5.

pMEZ12: $P_{lcpA}$-*lcpA* was amplified from genomic DNA of 630 strain using TC403/TC404 primers and inserted in pMEZ5 using the Golden Gate assembly protocol (NEB Biolabs) to give pMEZ12.

pJV20: $P_{lcpB}$-*lcpB* was amplified from genomic DNA of 630 strain using JV101/JV102 and inserted in pTC131 using the Golden Gate assembly protocol (NEB Biolabs) to give pJV20.

pJV21: pJV20 was digested using StuI and KpnI restriction enzymes, and the DNA fragment of approximately 1,5kb was ligated with pMTL84222 ($Erm^R$) previously digested by StuI and KpnI restriction enzymes.

pJV27: the $P_{tet}$ was amplified from pRPF185 using JV136/JV137 primers, and the *lcpB* was amplified from genomic DNA of 630 strain using JV138/JV139 primers. Both PCR products were inserted in pJV8 using the Gibson assembly protocol (NEB Biolabs) to give pJV27.

**Cloning *gusA* reporter plasmids**

pMDR1: Kanamycin cassette flanked with BsmBI restriction enzyme sites was amplified from pJV4 using TC393/TC394 primers. *gusA* was amplified from pRPF185 using TC395/TC396 primers. Both PCR DNA fragments were inserted into pTC131 using the Golden Gate assembly protocol to give pMDR1.

pMDR2: Spectinomycin cassette was amplified from pAT28 using TC397/TC398 and inserted into pMDR1 to give pMDR2.

pMDR8: $P_{lcpA}$ was amplified from genomic DNA of 630 strain using TC409/TC410 and inserted into pMDR2 to give pMDR8. $P_{lcpB}$ was amplified from genomic DNA of 630 strain using TC411/TC412 and inserted into pMDR2 to give pMDR5.
